# Integrating Hard Silicon for High-Performance Soft Electronics via Geometry Engineering

**DOI:** 10.1007/s40820-025-01724-1

**Published:** 2025-04-14

**Authors:** Lei Yan, Zongguang Liu, Junzhuan Wang, Linwei Yu

**Affiliations:** 1 School of Electronic Science and Engineering/National Laboratory of Solid-State Microstructures, Nanjing University, Nanjing, 210023 People’s Republic of China; 2 College of Physics Science and Technology/Microelectronics Industry Research Institute, Yangzhou University, Yangzhou, 225009 People’s Republic of China

**Keywords:** Soft electronics, Silicon, Geometry engineering, Silicon nanowires

## Abstract

Soft electronics, which are designed to function under mechanical deformation (such as bending, stretching, and folding), have become essential in applications like wearable electronics, artificial skin, and brain-machine interfaces. Crystalline silicon is one of the most mature and reliable materials for high-performance electronics; however, its intrinsic brittleness and rigidity pose challenges for integrating it into soft electronics. Recent research has focused on overcoming these limitations by utilizing structural design techniques to impart flexibility and stretchability to Si-based materials, such as transforming them into thin nanomembranes or nanowires. This review summarizes key strategies in geometry engineering for integrating crystalline silicon into soft electronics, from the use of hard silicon islands to creating out-of-plane foldable silicon nanofilms on flexible substrates, and ultimately to shaping silicon nanowires using vapor–liquid–solid or in-plane solid–liquid–solid techniques. We explore the latest developments in Si-based soft electronic devices, with applications in sensors, nanoprobes, robotics, and brain-machine interfaces. Finally, the paper discusses the current challenges in the field and outlines future research directions to enable the widespread adoption of silicon-based flexible electronics.

## Highlights

- Crystalline silicon (c-Si) is one of the most mature and reliable materials for high-performance electronic devices and has garnered widespread attention in the field of flexible electronics.
- This article examines the detailed transition enabled by geometry engineering from "3D bulk materials" to "2D thin films," and ultimately to "1D nanowires," highlighting the advancements and challenges in enhancing the flexibility and mechanical properties of c-Si.
- We emphasize the forefront applications of silicon nanowires in wearable electronics and healthcare, aiming to accelerate the rapid development of c-Si within the realm of flexible electronics.

## Graphical abstract

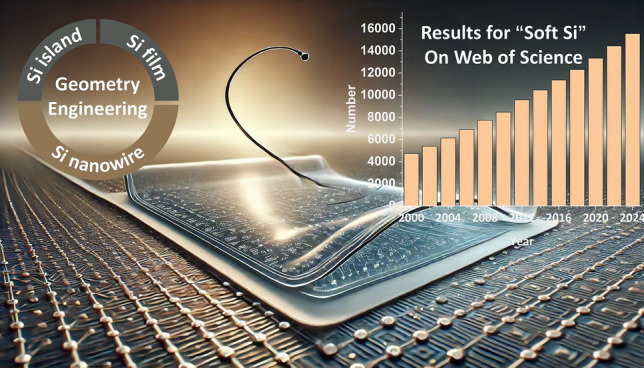

## Introduction

Soft electronics, also referred to as flexible electronics, encompass circuits and electronic components capable of maintaining functionality under conditions of bending, rolling, folding, or even stretching. This transformative technology has significantly reshaped the design and functionality of electronic devices [1–4]. Since their inception in the 1960s with the advent of flexible solar cells [5], the field has evolved dramatically, driven by advancements in material science and fabrication techniques. Compared to conventional solid-state electronics, flexible electronics present unique advantages, such as the ability to conform to non-planar surfaces and maintain performance under mechanical deformation [6–10]. These attributes make them indispensable for applications requiring lightweight, portable, and adaptable designs, particularly in areas like medical implants [11–15], wearable technology [16–21], and dynamic operational environment [22–25]. By leveraging these capabilities, flexible electronics enable innovative designs that prioritize user experience and comfort. This adaptability further facilitates the creation of more ergonomic and user-friendly devices, thereby expanding their potential applications.

With the rapid advancement of soft electronics, various flexible materials and structural designs have significantly enhanced the lightweight nature, mechanical flexibility, and portability of electronic devices. Numerous materials, such as organic semiconductors, two-dimensional materials, silicon, and others, have been investigated as substrates or functional components. Organic materials, for instance, offer excellent intrinsic flexibility and stretchability at a low cost; however, they often face limitations in electrical performance and long-term stability [26–30]. Moreover, many flexible electronic systems relying on macromolecular (polymer) chemistry are heavily dependent on fossil-derived resources like oil and natural gas. This dependence not only results in a substantial carbon footprint but also poses significant challenges to the long-term viability of polymer-based technologies as industries strive for low-carbon or zero-carbon frameworks by 2050 [31]. Similarly, while two-dimensional materials such as graphene and MoS_2_ exhibit excellent electrical and mechanical properties, their high production costs and energy-intensive synthesis methods limit their scalability and environmental compatibility [4, 32–36]. Against this backdrop, silicon-based flexible electronics emerge as a compelling alternative. Silicon (Si) is not only abundant in the Earth’s crust but also benefits from highly efficient and well-established manufacturing processes. These attributes position Si as a material capable of addressing both the performance and sustainability challenges of flexible electronics. This review delves into the potential of Si-based flexible electronics to meet global sustainability goals while delivering high reliability and superior functionality.

Silicon, a traditional semiconductor material, is now revealing its unique potential in flexible electronics, building upon its well-established applications in rigid electronics [6, 37–42]. Crystalline silicon (c-Si) offers several significant advantages: (1) c-Si possesses high electron mobility, making Si-based flexible electronic devices superior in speed and performance compared to many other flexible materials [43–46]. (2) c-Si exhibits stable operation under high current densities, a critical requirement for high-performance electronics [47–49]. (3) It maintains excellent stability at elevated temperatures and, compared to organic semiconductors, offers better chemical stability and environmental resistance, enabling it to function effectively in harsh environments [50–53]. These attributes make it suitable for flexible electronic applications in outdoor or extreme conditions. Furthermore, Si-based flexible electronics maintain stable performance over extended operational periods, enhancing their reliability in demanding applications. (4) Si is regarded as having good biocompatibility, meaning it does not provoke severe immune responses or toxicity [54, 55]. Biocompatibility is essential, as flexible electronics inevitably come into direct or indirect contact with the human body.

These features position Si as a key candidate material for high-performance, high-reliability flexible electronics. The growing demand for lightweight, portable, and adaptable electronic devices has spurred significant research and development in this area. However, the inherent rigidity of conventional wafer-scale c-Si presents challenges in its integration into flexible electronics, as stable electrical performance is required under mechanical stress or strain. To overcome these limitations, researchers have employed geometry engineering, which refers to a set of design and fabrication strategies that optimize the dimensions, structures, and morphologies of Si at micro- and nanoscale levels to enhance its mechanical flexibility. This includes techniques such as thinning, patterning, and nanostructuring, which reduce bending stiffness while largely preserving the intrinsic electrical properties of Si. Geometry engineering serves as a crucial approach among various strategies for integrating c-Si into flexible electronics, complementing other methods discussed in this review. Those approaches have proven critical for facilitating the seamless integration of c-Si into flexible electronic devices, paving the way for innovative applications in wearable electronics, biointerfaces, and beyond.

**Fig. 1:**
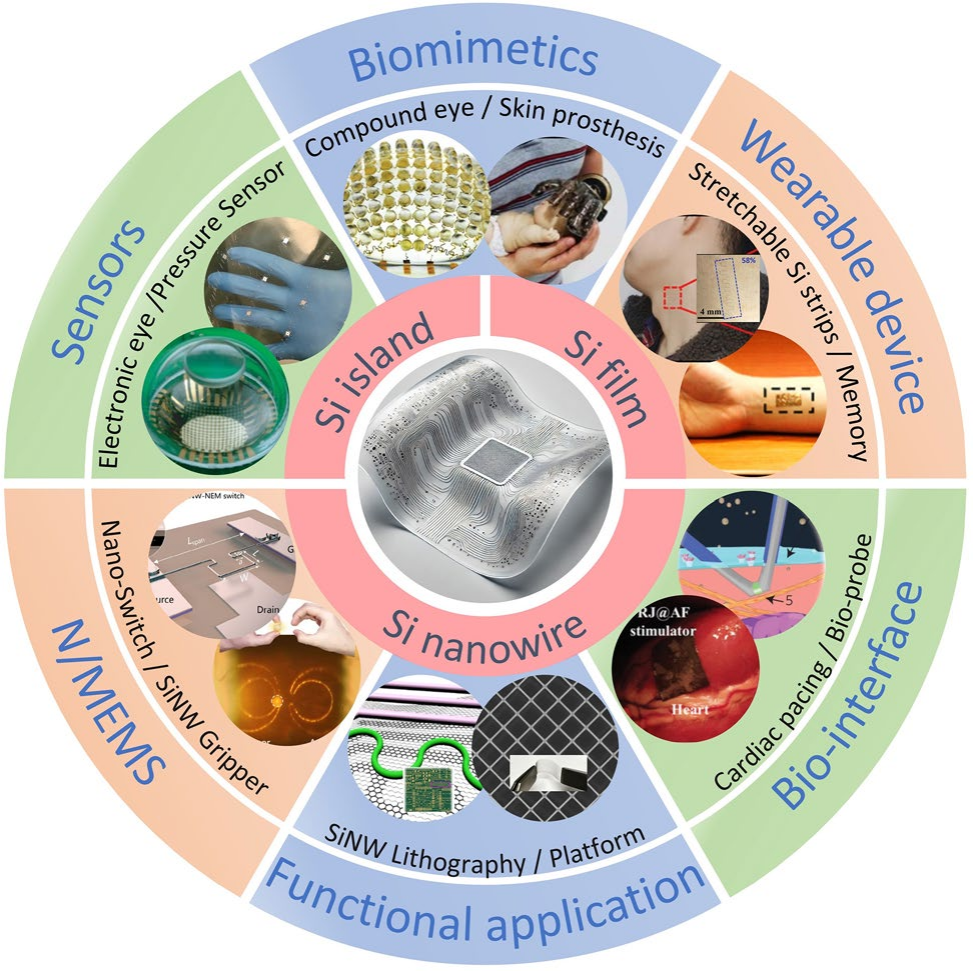
Main schemes to make crystalline silicon flexible and their applications in flexible electronics. Reproduced with permission from Ref. [16]. Copyright 2024, John Wiley and Sons. Reproduced with permission from Ref. [57]. Copyright 2016, Multidisciplinary Digital Publishing Institute. Reproduced with permission from Ref. [58]. Copyright 2008, J Springer Nature. Reproduced with permission from Ref. [59]. Copyright 2013, Springer Nature. Reproduced with permission from Ref. [60]. Copyright 2014, Springer Nature. Reproduced with permission from Ref. [61]. Copyright 2016, American Association for the Advancement of Science. Reproduced with permission from Ref. [62]. Copyright 2013, Springer Nature. Reproduced with permission from Ref. [63]. Copyright 2019, John Wiley and Sons. Reproduced with permission from Ref. [64]. Copyright 2023, Springer Nature. Reproduced with permission from Ref. [65]. Copyright 2024, American Chemical Society. Reproduced with permission from Ref. [66]. Copyright 2021, John Wiley and Sons. Reproduced with permission from Ref. [67]. Copyright 2019, Springer Nature

While several reviews have discussed the thinning of bulk Si for flexible applications [6, 37, 38, 56], there is still a gap in the literature regarding a comprehensive review of the transition from “3D bulk materials” to “2D thin films,” and finally to the geometry engineering of “1D nanowires”. Figure 1 illustrates the relevant design strategies and representative applications [16, 57–67]. Here we first introduce readers to the current three design strategies that impart mechanical flexibility to three-dimensional bulk c-Si through the “Si islands” design or thinning technology, and examine preparation strategies for c-Si nanowires in one-dimensional form, highlighting the mechanical flexibility and stretchability achieved through geometry engineering. Following this, we review the recent advances in the applications of flexible electronics based on silicon nanowires (SiNWs), such as sensors, bionics, wearable devices, biointerface engineering, and nano/microelectromechanical systems (N/MEMS), and other functional applications. Finally, we discuss the current challenges and future development prospects of Si-based flexible electronics.

## Fabrication Strategies of Flexible Silicon-Based Electronics

In response to the highly growing demand for electronic devices with wearable and portable functionalities, Si-based photovoltaic cells have been developed to be flexible, lightweight, and thin. In this section, we will comprehensively introduce the three fabricating technologies for flexible Si-based electronics, primarily focusing on island design of bulk Si materials, thin films from etching of Si wafer and geometry design of SiNWs.

### "Silicon Islands" Design

The “island protection” strategy in flexible electronics refers to safeguarding critical regions of electronic devices, typically composed of high-performance yet fragile materials, to maintain their functionality and structural integrity under mechanical stress, such as bending, stretching, or other deformations [68–71]. The main approaches for implementing Si islands involve several key strategies: (1) surface passivation and protective coatings, (2) structural design for stress mitigation, (3) optimizing the integration between the flexible substrate and silicon islands, and (4) flexible encapsulation techniques. For the Si islands protection, the small and hard silicon chips were assembled onto a flexible substrate, such as a polymer film and polydimethylsiloxane (PDMS) substrate [72–74]. The silicon devices were first fabricated on flexible substrates and then cut into small rectangular or other geometric shapes. The gaps between the islands allow the substrate to bend freely under mechanical stress without compromising the electronic performance of the Si islands.

**Fig. 2:**
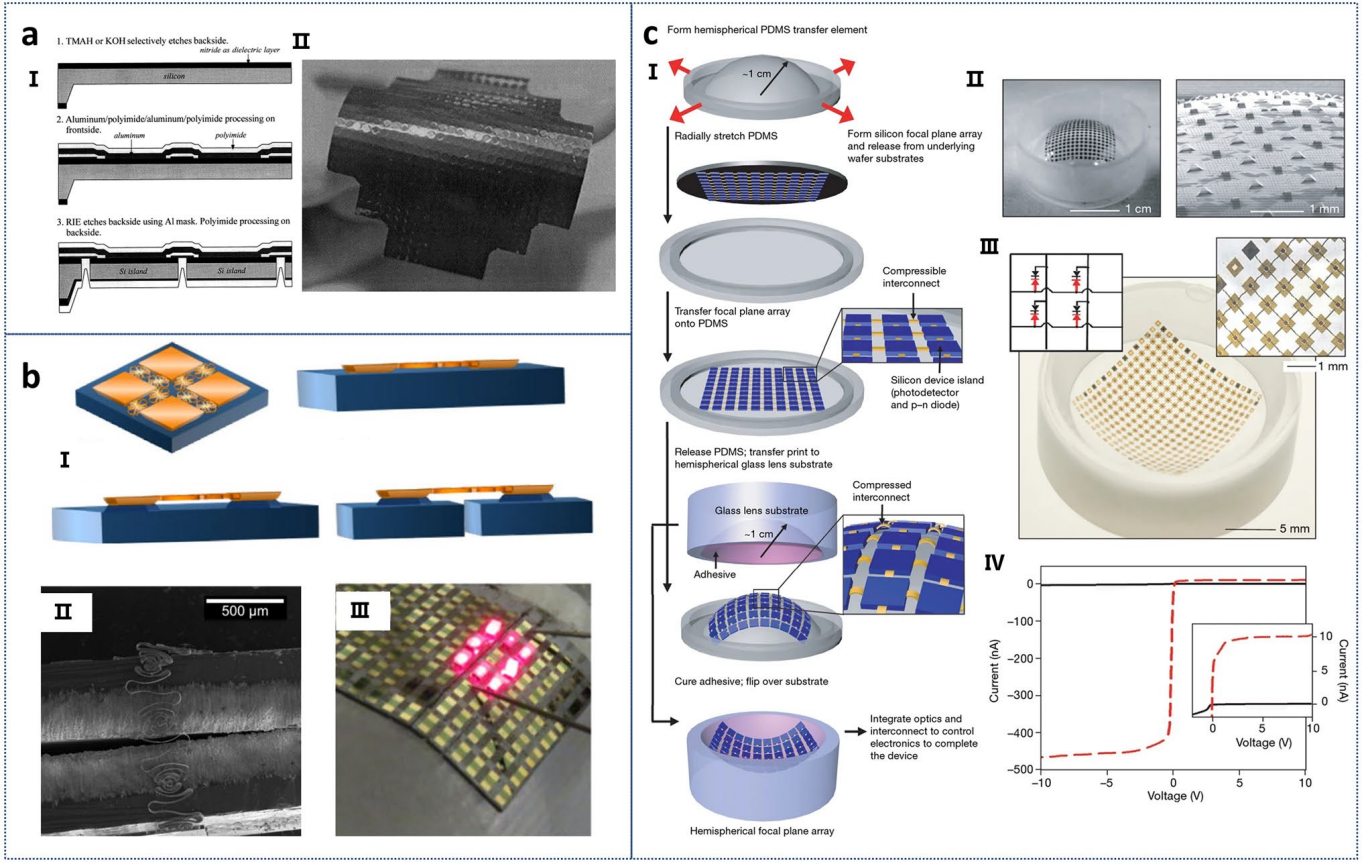
**a** I. A simplified process flow of flexible skin technology. II. Images of flexible skin at wafer scale. Reproduced with permission from Ref. [73]. Copyright 2000, Elsevier **b** I. Schematic diagram of the processing of silicon islands from split silicon wafers. II. The islands are folded downward, allowing the unpolished sides of the substrate to face each other. III. A flexible LED array. Reproduced with permission from Ref. [75]. Copyright 2017, AIP Publishing. **c** I. Fabrication process of an electronic eye camera with a compressible silicon focal plane array and a hemispherical PDMS transfer element. II. Photograph of the hemispherical PDMS transfer element with the focal plane array, SEM image of compressible interconnections, and the array integrated on a hemispherical glass substrate. III. Electrical characteristics showing high contrast response under light exposure with minimal leakage and reverse bias current. Reproduced with permission from Ref. [58]. Copyright 2008, J Springer Nature

In 1985, Barth and colleagues pioneered the batch fabrication of Si islands arrays on flexible polyimide substrates [72]. Their array, comprising 20 Si islands, each with a pn diode functioning as a thermometer, demonstrated a preliminary production yield. Jiang et al. applied silicon islands technology to shear stress sensors, developing a two-dimensional flexible skin with over 100 integrated sensors, as illustrated in Fig. 2a [73]. To further improve stretchability and foldability, Sepulveda et al. introduced a method for releasing silicon islands through fracture propagation (Fig. 2b-I, II) [75]. This technique enabled three-dimensional monolithic stacking of integrated circuits and other electronic devices without silicon vias and facilitated the development of wearable electronics compatible with flip-chip bonding (Fig. 2b-III).

Figure 2c shows the fabrication process of an electronic eye camera by Ko and colleagues, utilizing the Si islands approach (Fig. 2c-I) [58]. The design incorporated silicon device islands, including photodetectors and pn diodes, with electrical connections achieved through compressive interconnect structures (Fig. 2c-II, III). The entire array was mounted on a flexible hemispherical PDMS substrate, maintaining high electrical performance. Key performance characteristics included strong photoresponse, extremely low reverse bias current, and minimal pixel crosstalk in passive matrix addressing (Fig. 2c-IV). Overall, the Si islands strategy has been predominantly applied in the development of flexible sensing technologies.

In summary, the “island protection” strategy has been instrumental in addressing the rigidity of c-Si by segmenting it into discrete islands that maintain high electronic performance while allowing the flexible substrate to deform freely. Currently, this approach has enabled significant advancements in flexible sensing technologies, the “island protection” approach is not limited to Si but has also been extended to other materials used in flexible electronics [76–82]. However, the strategy still has some limitations, such as trade-offs between flexibility and protection, challenges in thermal management, and constraints in logic integration, which collectively restrict its application range.

These limitations primarily stem from the inherent mechanical and thermal constraints associated with the island-based structure. First, while the segmentation of Si into discrete islands reduces overall rigidity, the mechanical mismatch between the rigid islands and the soft substrate can lead to localized stress accumulation, potentially causing delamination or mechanical fatigue under repeated deformation [68, 83]. Second, thermal dissipation remains a critical challenge since heat generated within the silicon islands must be efficiently transferred through the surrounding flexible matrix, which often has lower thermal conductivity. This can lead to localized overheating, affecting device reliability [80, 84]. Additionally, the discrete nature of silicon islands poses integration challenges for complex logic circuits, as achieving reliable electrical interconnections across multiple islands requires additional design considerations [69, 85]. These drawbacks have motivated the exploration of alternative strategies, such as Si thinning, which offers improved flexibility, thermal management, and circuit integration capabilities.

### Thinning Silicon Wafer to Thin Films

Si thinning is an alternative approach to offer enhanced design flexibility and broader applicability for high-performance electronics. By significantly reducing the thickness of Si films, this approach greatly decreases bending stiffness while retaining silicon’s exceptional semiconductor properties, paving the way for innovative device designs.

The mechanical flexibility of silicon can be obviously enhanced by spatially reducing its thickness [37, 38, 86–89], while retaining its excellent electronic properties, such as high carrier mobility and low power consumption. Thin Si films are easier to integrate into flexible substrates, enabling electronic devices with high performance and flexibility [37, 90]. In addition, the stretchable Si thin film device can be realized by using prestrained substrates.

In the realm of flexible Si-based electronics, methods for fabricating c-Si films can be categorized into two distinct approaches: “top–down etching” and “bottom–up growth”, which, respectively, represent the strategy of processing bulk materials into thin films or gradually growing small-scale structures to achieve the desired film thickness. The top–down approach primarily involves the fabrication of thin films from existing single-crystal Si materials through physical or chemical means, making it suitable for applications requiring high-quality single-crystal Si films. However, it often entails higher costs and more complex processes. In contrast, the bottom–up approach directly deposits or grows c-Si films on a substrate, facilitating simpler processes for producing films on flexible substrates. Nevertheless, the quality of the crystal may be influenced by the substrate material and processing conditions. Reported top–down techniques include silicon-on-insulator (SOI) transfer technology [60, 91–95], < 111 > wafer undercut etching [96–100], crack-based exfoliation [101–105], and substrate removal etching [106, 107]. Bottom–up techniques encompass physical vapor deposition [108–110], chemical vapor deposition [111–113], and solid-phase epitaxy [114–118]. A comprehensive review of these techniques has been presented in previous literature [37, 38, 119].

**Fig. 3:**
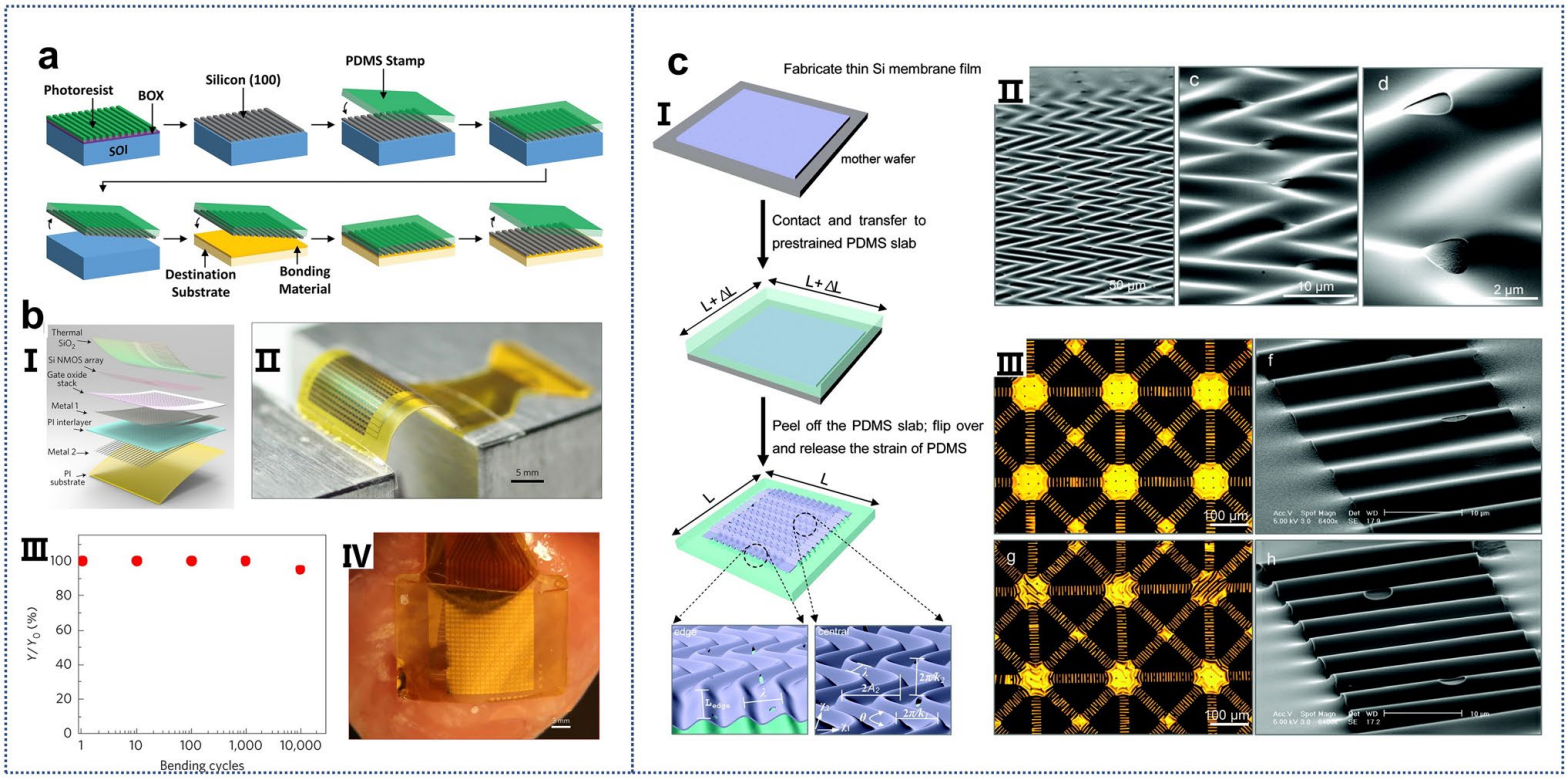
**a** Schematic illustration of the “SOI transfer” process. Reproduced with permission from Ref. [37]. Copyright 2015, John Wiley and Sons. **b** I. Exploded view of the capacitive coupling flexible sensing system. II. Images of the devices during mechanical bending tests. III. Yield versus bending cycles under a 5 mm radius, showing minimal change over 10,000 cycles. IV. Photograph of the sensing system on a Langendorff-perfused rabbit heart. Reproduced with permission from Ref. [128]. Copyright 2017, J Springer Nature. **c** I. Fabrication steps for a 2D "wavy" semiconductor nanomembrane on an elastic support. II. SEM image of the wavy Si nanomembrane on PDMS. III. Optical image of the wavy structure designed for edge effects and 2D stretchability in a flat island array. Reproduced with permission from Ref. [132]. Copyright 2007, American Chemical Society

One of the most common methods for obtaining ultrathin Si films is through the exfoliation of SOI substrates. Figure 3a illustrates the general process of acquiring Si films on flexible substrates, which involves selectively etching buried oxide layers with hydrofluoric acid (HF), followed by the transfer of the top Si layer to the flexible substrate to form devices [37]. This process has been extensively studied in the literature, where Si microstrips have been obtained from SOI wafers and transferred to flexible substrates for the fabrication of logic transistors and radio frequency components [120–127]. Figure 3bI–IV depicts the flexible Si transistor capacitive coupling arrays fabricated by Fang et al. using this technology on plastic substrates, demonstrating good performance after over 10,000 bending cycles [128]. By eliminating all direct metal interfaces and replacing them with capacitive sensing nodes integrated into a high-performance, flexible Si electronics platform, a robust and scalable solution has been provided for system electrophysiological studies in normal, pacing, and arrhythmia conditions in Langendorff hearts, thereby offering a practical pathway for the widespread application of biocompatible flexible electronic implants.

Transistors are essential for information processing and equally important for data storage. Kim et al. introduced a wearable, fully multiplexed silicon non-volatile memory array featuring nanocrystal floating gates [61]. The uniform nanocrystal charge traps significantly enhance the storage window margin and retention performance. Furthermore, based on ultrathin Si nanofilm circuits, the combination of memory multiplexing with sensor signal amplification enables wearable medical applications, such as the long-term storage of heart rate monitoring data. Building on the concept of thinned silicon substrates, Zhang et al. developed a novel silicon microstructure—a monocrystalline Si framework by combining wet etching with micromachining techniques [66]. This framework exhibits self-supporting, flexible, lightweight, customizable, and highly transparent. Benefiting from its hollow frame structure, it can withstand small bending radii of less than 0.5 mm, achieving transparency of up to 96% across all wavelengths. The monocrystalline silicon framework provides a new platform for high-performance transparent flexible optoelectronics.

While single ultrathin Si films demonstrate good mechanical flexibility, stretchability is equally important. To achieve enhanced stretch performance, geometry engineering strategies for stretchable single-crystal Si have been proposed [129–132]. The stretchable single-crystal Si shown in Fig. 3c comprises a two-dimensional bending and “wavy” Si nanomembrane on an elastic substrate [132]. Figure 3c-I presents a schematic of the steps involved in fabricating a two-dimensional “wavy” semiconductor nanomembrane on an elastic substrate, where the pre-strained geometric morphology imparts stretchability to the Si film. Choi et al. described various aspects of this geometry and the response to uniaxial and biaxial strains in different directions, facilitating the realization of high-performance electronic devices with full two-dimensional stretchability (Fig. 3c-II and III).

**Fig. 4:**
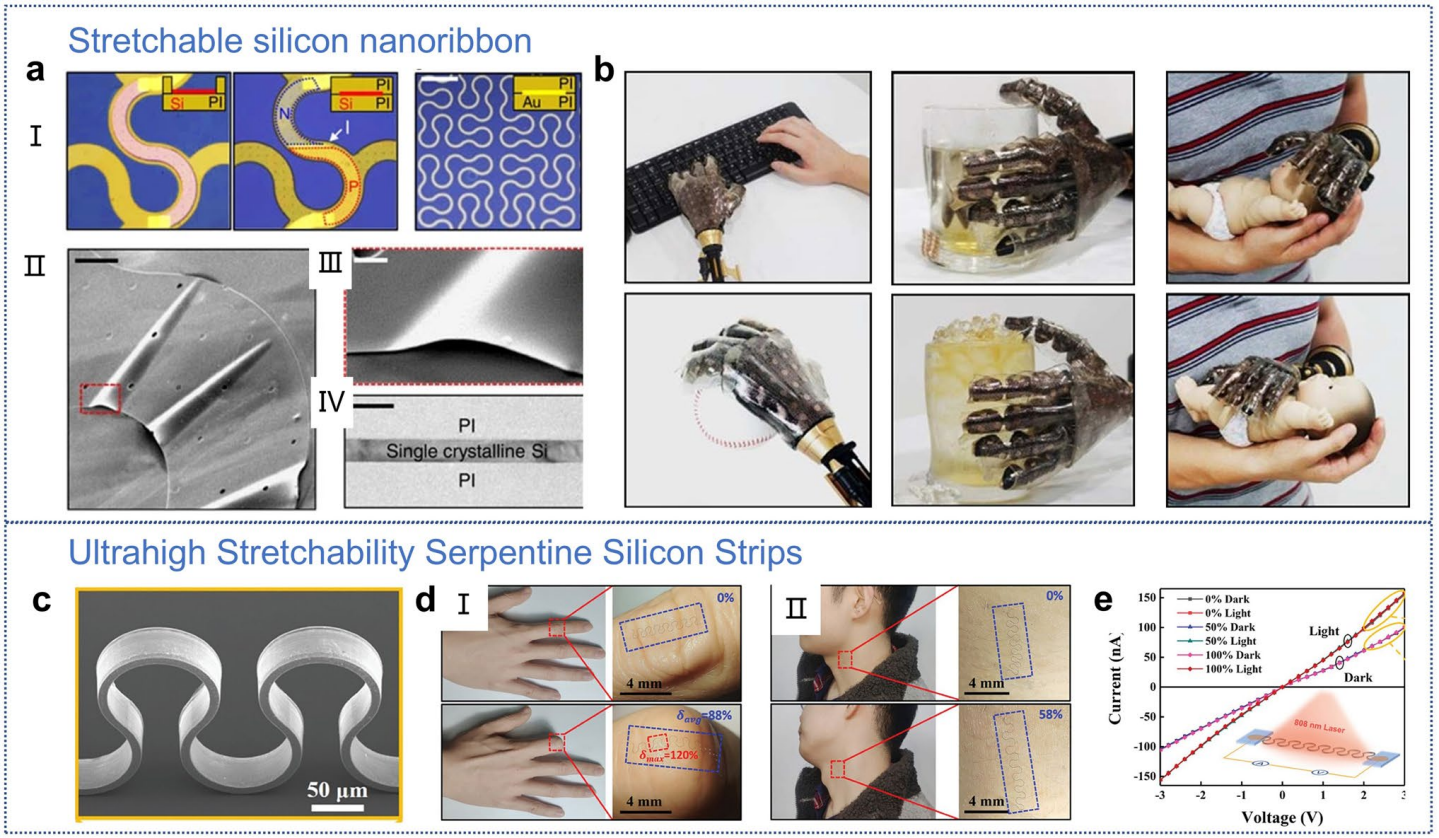
**a** I. Micrographs of ultrathin single-crystal silicon nanoribbons (SiNR) as pressure and temperature sensors. II. SEM image of SiNR on a silicon dioxide substrate. III. Wrinkle formation demonstrating SiNR flexibility. Scale bar: 20 μm. IV. Enlarged view of wrinkled SiNR. Scale bar: 2 μm. V. Cross-sectional TEM image of the strain gauge with SiNR encapsulated in a PI layer at the neutral mechanical plane. Scale bar: 200 nm. **b** Images of a prosthetic hand: typing on a keyboard, catching a baseball, interacting with hot and ice water, and touching the head and abdomen of a baby doll. **a, b** Reproduced with permission from Ref. [60]. Copyright 2014, Springer Nature. **c** SEM images of highly stretchable serpentine silicon strips (FS-Si strips). **d** Photographs of FS-Si strips adhered to a finger joint and neck. **e** I–V curves of FS-Si photodetectors under 0%, 50%, and 100% strain, in darkness and under 808 nm illumination. **c–e** Reproduced with permission from Ref. [16]. Copyright 2024, John Wiley and Sons

Based on the design of pre-strained morphology, subsequent work by Kim et al. reported a stretchable prosthetic skin equipped with an array of ultrathin single-crystal silicon nanoribbon (SiNR) strain, pressure, and temperature sensors (Fig. 4a) [60]. The geometry of the SiNR sensor array can be adjusted according to the dynamic mechanical properties of the target skin area for stretchability. This design strategy provides the highest levels of spatiotemporal sensitivity and mechanical reliability, greatly enhancing the artificial skin’s ability to perceive highly variable external environments. The integration of stretchable humidity sensors and heaters further enables the sensing of skin moisture and body temperature regulation. Corresponding electrical stimuli can be transmitted from the prosthetic skin to the body, stimulating specific nerves through conformal contact with ultrathin stretchable nanowire electrodes (Fig. 4b). In pursuit of even more extreme stretch performance, Shi et al. demonstrated an independent serpentine Si ribbon with an ultra-high stretchability (over 300%), albeit at the cost of integration [16]. The serpentine Si ribbon exhibits excellent stability and durability over 50,000 cycles of 100% stretching (Fig. 4c). Wearable devices can maintain conformal contact with the skin, achieving a maximum stretchability of 120% (Fig. 4d). They are electrically insensitive to stretching, ensuring signal stability during everyday use (Fig. 4e). By combining mature processing technologies with the excellent semiconductor properties of silicon, serpentine Si ribbons are poised to become a core stretchable electronic material for wearable electronics.

In summary, the thinning of Si films plays a crucial role in enabling flexible electronics, offering not only enhanced mechanical flexibility but also improvements in electrical performance, thermal management, and overall device robustness. Both top–down and Bottom–up approaches have been extensively explored, with each method presenting its own advantages and limitations. While top–down techniques, such as SOI transfer and wet etching, produce high-quality c-Si films suitable for advanced applications, they often involve complex and costly processes. Conversely, Bottom–up techniques like chemical vapor deposition (CVD) provide more scalable solutions but can face challenges related to crystal quality. Despite these trade-offs, silicon’s superior semiconductor properties, combined with innovative structural designs like wavy nanomembranes and serpentine Si strips, have made it an integral material in the development of high-performance stretchable electronics. However, the quest for even greater flexibility and improved integration has led to the exploration of SiNWs, which hold promise for further enhancing device performance.

### Geometry Design of Silicon Nanowires

The flexibility of Si can be further enhanced when its size is down to nanoscale diameters. The 1D SiNWs exhibit a high aspect ratio, excellent electrical properties, high sensitivity, and exceptional mechanical flexibility, making them promising candidates for applications in flexible electronic devices. Additionally, their compatibility with existing Si-based processes enhances their potential for practical applications. More importantly, these SiNW are easily designed to wavy and kinked shapes through geometry engineering, which offer superior stretchability when compared to the previous two approaches [133–137]. In this section, we will delve into how the unique properties of SiNWs, including their nanoscale geometry and excellent electrical characteristics, make them an ideal candidate for next-generation flexible electronic applications. Firstly, we will discuss the manufacturing methods of SiNWs, which can be mainly classified into two categories: top–down, bottom–up.

#### Top–Down

Top–down approaches generally offer higher yield and device-to-device reproducibility, which is favorable for large-scale production. These methods involve lithography, etching, and further material deposition steps, resulting in high-density devices with precise designs and positions, a critical requirement in computing technologies. Several lithography techniques, such as electron beam lithography (EBL), focused ion beam (FIB), nanoimprint lithography, and local oxidation nanolithography using atomic force microscopy, allow for the well-ordered arrangement and geometric design of SiNWs [138–143]. However, challenges like the need for high-resolution, complex equipment and reliance on SOI wafers often lead to higher fabrication costs.

Another top–down approach is Metal-Assisted Chemical Etching (MACE), a cost-effective method to produce SiNWs [144–148]. In MACE, a noble metal catalyst (e.g., silver or gold) facilitates selective etching of Si in an oxidizing and etching chemical solution. This method allows for the fabrication of vertically aligned SiNWs directly from bulk silicon wafers. The advantages of MACE include its simplicity, scalability, and compatibility with large-area Si substrates, making it particularly suitable for high-throughput production. Moreover, the process can achieve high aspect ratios and maintain the crystallinity of silicon, which is essential for electronic applications. However, MACE also has limitations, including challenges in precisely controlling the nanowire diameter and distribution, as well as the lack of capability to design nanowire morphologies, which is crucial for improving their flexibility and stretchability.

#### Bottom–Up

**Fig. 5:**
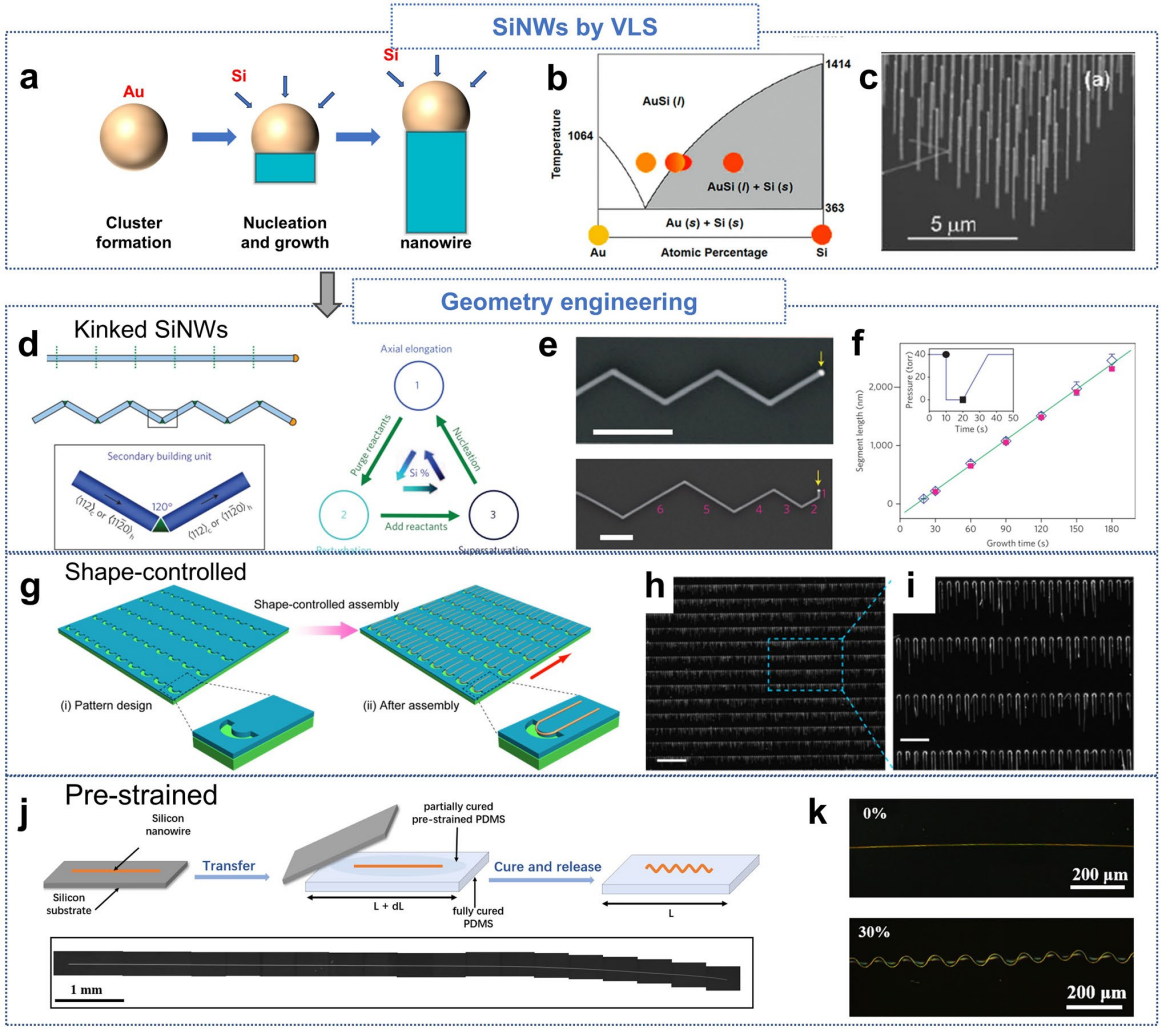
**a** Schematic illustration of Si nanowire growth via the VLS mechanism. **b** Binary phase diagram of the AuSi system. Reproduced with permission from Ref. [159]. Copyright 2006, Institute of Physics Publishing.** c** SEM image of SiNWs grown using the VLS method (30° tilt). Reproduced with permission from Ref. [160]. Copyright 2011, American Chemical Society. **d** Schematic of coherent kinked nanowires and secondary building units (SBUs) with stepwise synthesis cycles.** e** SEM images of multi-kinked 2D SiNWs with equal (top) and reduced arm lengths (bottom). Scale bar: 1 μm. **f** Graph showing the relationship between segment length and growth time. **d–f** Reproduced with permission from Ref. [171]. Copyright 2009, Springer Nature. **g** Schematic of U-shaped nanowire assembly with SEM images of U-shaped SiNWs in trenches. Scale bars: **h** 200 μm; **i** 50 μm. **g–i** Reproduced with permission from Ref. [174]. Copyright 2016, American Chemical Society.** j** Schematic of the fabrication process for stretchable SiNWs. **k** Optical microscope images of stretchable SiNWs with prestrains of 0% (top) and 30% (bottom). **j, k** Reproduced with permission from Ref. [176]. Copyright 2020, American Chemical Society

In Bottom–up fabrication, atoms assemble into the desired structures, typically using CVD with SiH_4_ via the vapor–liquid–solid (VLS) mechanism [149–155]. A metal nucleation catalyst (e.g., gold nanoparticles) forms a liquid metal–semiconductor eutectic alloy at high temperatures. Continuous supply of semiconductor material at the vapor–liquid interface leads to supersaturation, followed by precipitation of the semiconductor at the liquid–solid interface, driving nanowire growth [152, 156–158]. The diameter of the resulting SiNWs can be controlled by adjusting the size of the catalyst droplets and the liquid–solid interface, as illustrated in Fig. 5a, b [159]. The VLS mechanism offers precise control over nanowire diameter and allows for in situ doping, maintaining high crystallinity throughout the process. Bottom–up-grown nanowires can achieve extremely small diameters (as thin as 3 nm) and allow large-scale growth in a single process, as shown in Fig. 5c [160]. However, the use of gold introduces a critical issue of potential contamination. Gold atoms can diffuse into the silicon matrix, acting as deep-level traps that degrade carrier mobility and increase leakage currents, particularly in electronic devices relying on high-quality semiconductors [161].

This contamination is especially significant in high-performance devices such as field-effect transistors (FETs), where carrier transport properties directly impact functionality. Alternative catalysts, such as indium [162, 163], aluminum [164], or copper [165, 166], have been explored to reduce contamination risks while maintaining growth efficiency. For instance, indium-based catalysts can facilitate the VLS process at relatively low temperatures, potentially mitigating the formation of deep-level traps. Nevertheless, these alternative materials often involve trade-offs, such as reduced control over nanowire morphology or limitations in scalability. To address these challenges, research focuses on optimizing the growth environment to minimize contamination. Strategies include the use of surface passivation layers to prevent catalyst diffusion [167, 168] and the exploration of gold-free catalytic processes, such as catalyst-free growth [169]. These approaches aim to enhance the compatibility of SiNWs with sensitive electronic applications while preserving the advantages of the VLS mechanism.

While these methods offer substantial flexibility and scalability, the mechanical flexibility and stretchability of SiNWs can be further enhanced through advanced geometry engineering techniques. By manipulating the nanowires’ morphology, it is possible to transition from straight to more complex, curved structures, thereby improving their mechanical properties, especially in terms of stretchability. One promising approach involves a “nanotectonic” technique that enables the growth of kinked or zigzag SiNWs [62, 170–173], where straight sections are separated by triangular joints (Fig. 5d–f). This method allows precise control over the growth of the nanowires, including modulation of doping profiles for applications like field-effect transistors and p–n junctions at the kinked junctions, contributing to the versatility of SiNWs.

Another approach, known as guided shaping, involves lithographically patterning U-shaped trenches on a substrate (Fig. 5g) [174, 175]. Nanowires are assembled within these grooves, creating large-scale arrays with high-precision shape control (Fig. 5h, i). The curvature and mechanical properties of these U-shaped SiNWs can be optimized for integration in flexible circuits, facilitating improved mechanical performance without compromising electrical properties.

Finally, pre-straining SiNWs on an elastic substrate provides an effective strategy to induce stretchable configurations and enhance mechanical resilience (Fig. 5j). [176, 177] For example, ultraminiaturized, stretchable strain sensors have been developed using centimeter-long SiNWs, achieving a remarkable strain sensing range and durability. The pre-straining process introduces wavy or serpentine morphologies (Fig. 5k), which allow these nanowires to stretch without mechanical failure, a key feature in wearable electronics.

**Fig. 6:**
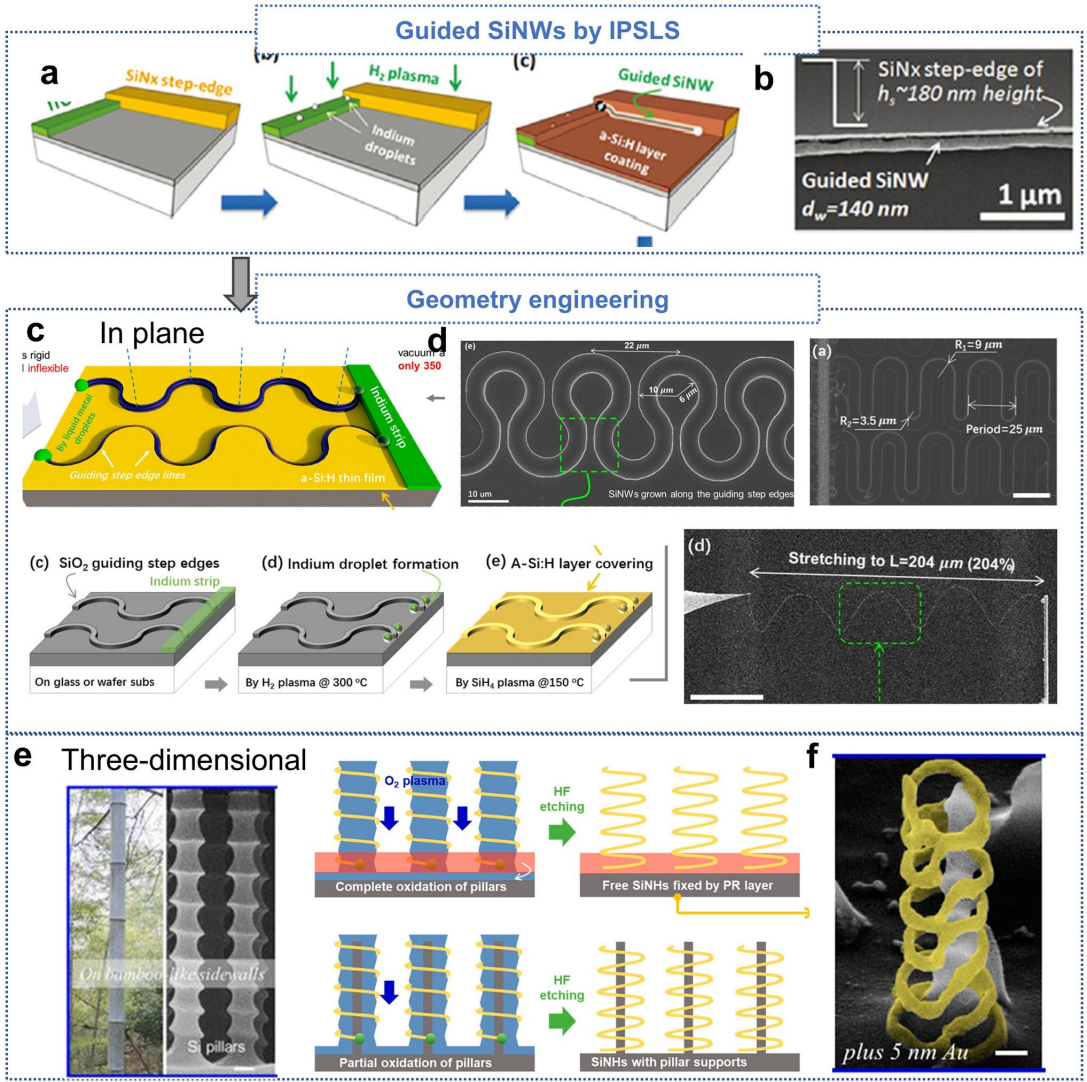
**a** Schematic illustration of the process for fabricating guided SiNWs by IPSLS. **b** SEM image of SiNWs growing along the guiding edges. **a, b** Reproduced with permission from Ref. [180]. Copyright 2015, Royal Society of Chemistry. **c** Schematic of in-plane SiNWs growth along wavy step edges. **d** Geometry line-shape engineering of planar SiNWs, including wavy and U-shaped structures. Reproduced with permission from Ref. [183]. Copyright 2017, American Chemical Society. **e** Schematic of helical nanowire growth on bamboo-like cylindrical structures. **f** Freestanding helical structure formed by the release of SiNHs, with a scale bar of 200 nm. Reproduced with permission from Ref. [184]. Copyright 2020, American Chemical Society

In-plane solid–liquid–solid (IPSLS) growth, much like VLS growth, involves the assembly of atoms to form SiNWs. However, IPSLS differs significantly in that SiNWs grow laterally along a substrate, transitioning from the vertical growth characteristic of VLS to a horizontal plane [178, 179]. This method enables SiNWs to be guided along lithographically defined channels (Fig. 6a, b) [180, 181]. A major advantage of IPSLS over top–down techniques is its ability to facilitate large-scale SiNW fabrication without requiring high-resolution equipment or complex processing. IPSLS can achieve the large-scale fabrication of SiNWs with relatively low-precision equipment. Moreover, in contrast to Bottom–up approaches that often necessitate additional strategies to ensure uniform nanowire alignment, IPSLS allows for straightforward integration and precise positioning of SiNWs on planar substrates. This technique is particularly advantageous in terms of cost and scalability, aligning with standard Si-based processes and making it highly practical for industrial applications.

Furthermore, IPSLS offers significant flexibility in shaping the SiNWs [64, 65, 182]. Growth-guiding trenches enable precise control over SiNW morphology, allowing for structures ranging from straight to intricate geometries (Fig. 6c) [183]. This morphological control eliminates the need for external forces to bend or shape the nanowires, thereby reducing stress accumulation that can compromise mechanical performance (Fig. 6d). Additionally, IPSLS enables the three-dimensional growth of SiNWs, such as spiral or helical structures, which further enhances their mechanical flexibility and enables applications that require stretchability.

Incorporating geometry engineering through IPSLS, researchers have successfully demonstrated the deterministic growth of SiNWs into predefined shapes, such as highly stretchable springs or intricate 2D patterns. For example, by using indium droplets to guide the absorption of amorphous Si precursor films, ultralong crystalline SiNWs have been grown along programmed step edges with excellent monocrystalline quality. This enables the fabrication of highly elastic SiNW structures that can withstand significant stretching deformations while preserving excellent electrical conductivity. Similarly, three-dimensional helical SiNWs growth have been achieved by leveraging corrugated grooves on bamboo-like cylinders, enabling precise control over the nanowires’ diameter, pitch, and symmetry (Fig. 6e, f) [184]. These innovations highlight the potential of IPSLS in constructing sophisticated, high-performance, and stretchable nanowire-based electronics.

In summary, SiNWs have emerged as one-dimensional nanostructures with unique properties, including exceptional mechanical flexibility, high sensitivity, and low power consumption. Their compatibility with established silicon-based processes further elevates their potential for flexible electronics. Various fabrication methods, such as top–down, bottom-up, and IPSLS techniques, have been developed to enable the precise control of SiNWs growth and integration. Top–down methods excel in yield and reproducibility, while Bottom–up approaches, particularly those based on VLS mechanisms, offer fine control over nanowire diameter and enable high crystallinity. IPSLS provides a cost-effective alternative by facilitating lateral SiNWs growth on planar substrates, allowing for guided morphological designs and ease of large-scale integration. These methods, coupled with advanced geometry engineering techniques, have paved the way for innovative SiNWs applications in flexible and stretchable electronics.

To provide a systematic comparison of different structural design and geometry engineering strategies, we have summarized their advantages, disadvantages, flexibility enhancement techniques, and sensitivity in strain sensors in Table 1. These strategies can be broadly categorized into three main approaches: silicon island design, silicon thinning, and silicon nanowire integration. Silicon island structures utilize segmented rigid components with flexible interconnects, balancing mechanical robustness with electronic performance. Silicon thinning techniques enhance flexibility by reducing bending stiffness while achieving higher integration density, though they often involve complex fabrication processes. Meanwhile, silicon nanowires offer exceptional mechanical compliance and high strain sensitivity due to their nanoscale dimensions and piezoresistive effects. Table 1 provides a comparative summary of these approaches, highlighting their key performance characteristics, representative techniques, and relevant works.

**Table 1:** Comparison of structural design and geometry engineering strategies

Strategy	Flexibility	Sensitivity in strain sensors (Reasons for high sensitivity)	Advantages	Disadvantages	Flexibility enhancement techniques	Representative works
Silicon island design	Medium (Segmented rigid structure)	Medium (Strain is localized at interconnects)	Maintains high electronic performance; Compatible with CMOS processes; Provides mechanical protection to key functional components	Restricted strain; Low integration density	Serpentine interconnects	[69, 75]
					Elastomer embedding, stretchable substrates, etc	[58, 73]
Silicon thinning	High	High (Thin structure allows for greater mechanical deformation)	Reduces bending stiffness; Enhances device integration; Retains excellent semiconductor properties; Enables large-area flexible electronics	Complex fabrication process; High cost	Wavy/Nanomembrane silicon	[129–132]
					Serpentine structures, etc	[16, 60, 185]
Silicon nanowires	Very high	Very high (High surface-to-volume ratio, piezoresistive effect)	Outstanding flexibility; Superior sensitivity in strain sensors due to piezoresistive effects; High adaptability in stretchable electronics	Lower carrier mobility compared to bulk silicon	Kinked SiNWs, Pre-straining SiNWs,	[62, 170–172]
					Pre-straining SiNWs,	[174–177]
					Geometric SiNWs via IPSLS Growth, etc	[64, 65, 182–184]

Understanding the mechanical properties of these nanostructures is essential for evaluating their practical performance in flexible electronics. In the following section, we discuss the deformation strain, superplasticity, and fracture toughness of nanostructured silicon to further illustrate its mechanical reliability under various conditions.

### Mechanical Properties of Nanostructured Silicon

The mechanical properties of nanostructured silicon play a critical role in its application within flexible electronics. Key aspects such as deformation strain, superplasticity, and fracture toughness directly influence the reliability, stretchability, and overall performance of devices under various mechanical stresses. This section provides an in-depth analysis of these mechanical characteristics, complemented by a summary table for clarity.

#### Deformation Strain of Silicon Nanostructures

Deformation strain is a critical parameter for evaluating the mechanical flexibility of silicon nanostructures. Bulk crystalline Si is inherently brittle, with a fracture strain of approximately 1% under tensile stress [186]. However, when scaled down to nanostructures such as nanowires, thin films, or membranes, Si demonstrates enhanced flexibility due to the relaxation of strain energy and suppression of defect propagation. SiNWs can sustain tensile strains over 10% before failure, a remarkable enhancement compared to bulk Si [187].

This enhancement arises from size-dependent plasticity, where dislocation motion is constrained, and surface effects dominate the mechanical response [188–190]. For instance, SiNWs with diameters below 50 nm exhibit a “smaller is stronger” behavior, where reduced size leads to increased strength and strain tolerance [191]. These properties make SiNWs ideal candidates for applications requiring high mechanical reliability under repetitive bending or stretching.

#### Superplasticity in Nanostructured Silicon

Superplasticity, defined as the ability of a material to undergo large, uniform plastic deformation, has been observed in silicon nanostructures under specific conditions. Unlike bulk Si, which lacks significant plasticity, nanostructures such as nanowires and thin films demonstrate superplastic behavior when subjected to high temperatures and slow strain rates. This phenomenon is primarily attributed to enhanced grain boundary sliding and diffusional creep mechanisms at the nanoscale.

The ability of nanostructured Si to deform plastically without fracturing under specific conditions provides new opportunities for integrating Si into flexible and stretchable electronics, particularly in harsh environments [171, 174, 187].

#### Fracture Toughness and Fatigue Resistance

Fracture toughness is another crucial metric for evaluating the mechanical robustness of nanostructured silicon. While bulk Si exhibits low fracture toughness (~ 0.7 MPa m^1/2^) [192], nanostructuring significantly improves this property. Experiments show that SiNWs and thin Si films exhibit enhanced fracture toughness, attributed to the suppression of microcrack initiation and propagation at the nanoscale [129, 193].

Additionally, fatigue resistance, or the ability to withstand cyclic loading without failure, is a key consideration for wearable electronics. SiNWs and other nanostructures demonstrate excellent fatigue resistance due to their ability to dissipate energy through nanoscale deformation mechanisms. For example, cyclic bending tests on silicon nanomembranes have shown minimal degradation in mechanical performance even after 10,000 cycles [128].

Table 2 summarizes the key mechanical properties of nanostructured Si, highlighting their advantages over bulk Si. These properties not only enable improved mechanical reliability but also expand the application scope of Si in flexible and stretchable electronic devices.

**Table 2:** Comparison of mechanical properties of bulk Si and nanostructured Si

Property	Bulk silicon	Nanostructured silicon	Remarks
Fracture strain	~ 1%	> 10% (SiNWs)	Enhanced flexibility due to size-dependent plasticity
Superplasticity	Absent	Observed at high temperatures	Enabled by grain boundary sliding and diffusional creep
Fracture toughness	~ 0.7 MPa m^1/2^	Higher (varies with size)	Suppression of crack propagation at nanoscale
Fatigue resistance	Low	High	Demonstrates durability under cyclic mechanical loading

In summary, nanostructured Si exhibits superior mechanical properties compared to bulk Si, including enhanced deformation strain, superplasticity, and fracture toughness. These characteristics are critical for the design and fabrication of high-performance flexible electronics, ensuring mechanical reliability and durability under complex loading conditions. Future research should focus on optimizing these properties through advanced nanofabrication techniques and exploring their integration into multi-functional devices.

## Advanced Applications of Flexible Si-Based Electronics

SiNWs with their unique properties such as high surface-to-volume ratio, excellent electrical conductivity, and mechanical flexibility, offer immense potential for a wide range of applications. Their compatibility with silicon-based processes further enhances their integration into advanced technologies. In this chapter, we will explore the applications of SiNWs across several key areas: sensing, bionics, wearable devices, biointerface engineering, and nano/microelectromechanical systems (N/MEMS). Each of these domains benefits from the inherent properties of SiNWs, enabling the development of innovative devices with enhanced performance and novel functionalities. Through a combination of nanostructure engineering and precise control over SiNWs growth, the versatility of SiNWs can be harnessed to meet the growing demands of next-generation flexible electronics and bioelectronics. The following sections will provide a detailed discussion on the application of SiNWs in these fields, highlighting their role in addressing technical challenges and advancing technological frontiers.

### Flexible Transistors

In the realm of flexible electronics, transistors serve as essential components responsible for crucial functions, such as signal amplification, switching control, system integration, and energy efficiency optimization. Their performance directly influences the response speed, power consumption, and overall stability of devices. SiNWs exhibit unique advantages in flexible transistors, with their high electron mobility and exceptional electrical properties significantly enhancing signal processing capabilities. The diminutive size and remarkable flexibility of SiNWs ensure seamless integration with flexible substrates, while the mature processing techniques associated with Si further bolster their performance in low-power applications. Consequently, SiNW-based flexible transistors provide not only outstanding electrical performance but also long-term stability and durability, propelling the advancement of flexible electronic devices toward higher performance, lower power consumption, and multi-functionality.

**Fig. 7:**
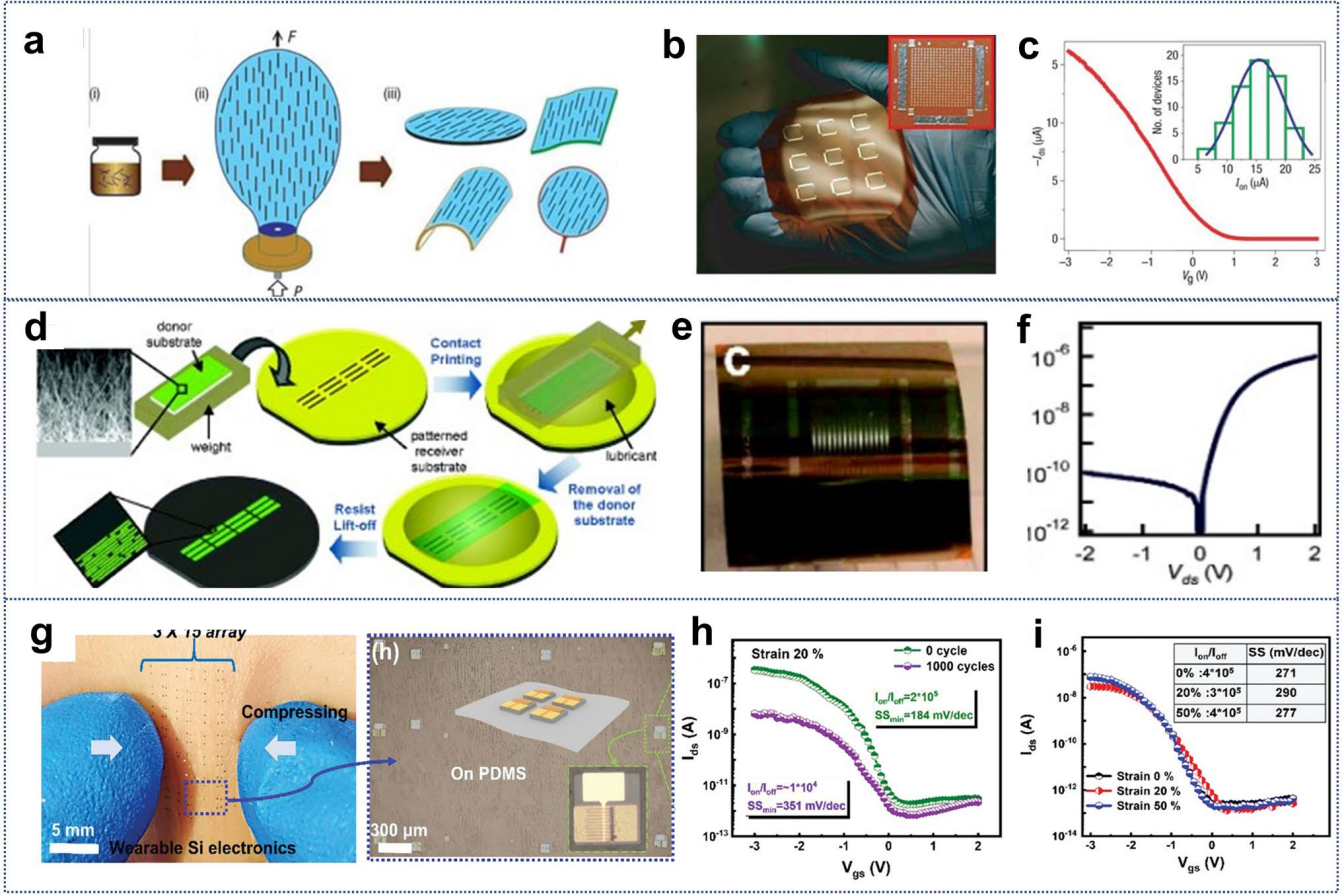
**a** Large-scale wafer-sized SiNWs FET arrays on plastic substrates using uniformly aligned SiNWs through the bubble-blowing transfer method. **b** Flexible FET array. **c** Transfer characteristic curve. **a–c** Reproduced with permission from Ref. [198]. Copyright 2007, Springer Nature. **d** Wafer-level assembly of highly ordered silicon nanowire arrays by contact printing. **e** Optical photograph of p-SiNWs on a flexible plastic substrate.** f** *I − V* characteristic of a representative SiNW Schottky diode. **d–f** Reproduced with permission from Ref. [204]. Copyright 2008, American Chemical Society. **g** The stretchable FETs constructed onto the soft PDMS substrates, with the protection of hard SiO_2_ island. **h, i** Transfer characteristics curves measured at different strain of 0%, 20%, 50% and at strain 20% over 1000 cycles. **g–h** Reproduced with permission from Ref. [205]. Copyright 2022, John Wiley and Sons

For transistors fabricated from SiNWs via VLS growth method, achieving uniform device performance necessitates the creation of ordered arrays of nanostructures. This requirement complicates the encapsulation of functions within individual nanowires, necessitating sophisticated techniques for alignment and assembly. Various methods have been proposed, including contact printing technique [194], magnetic field alignment [195, 196], fluid alignment [197, 198], alternating electric fields [199], holographic optical traps [200], dielectrophoresis [201], Langmuir–Blodgett techniques [202], and nanocombing [203]. For instance, Yu et al. employed a bubble-film transfer method to address the need for large-area, uniform arrangement and controllable density of SiNWs, efficiently fabricating large-wafer-scale SiNWs field-effect transistor (FET) arrays on plastic substrates (Fig. 7a–c) [198]. The FETs demonstrated excellent uniformity with an on-current of 15.1 ± 3.7 µA and a threshold voltage of 0.81 ± 0.32 V, attributed to the consistent density, alignment, and preferential distribution of nanowires on the bubble film’s surface.

Fan et al. showcased a simple contact printing technique that enabled the assembly of highly uniform, repeatable, and densely ordered nanowire arrays at the wafer scale (Fig. 7d–f). [204] This meticulous assembly control was achieved through the application of lubricants during the contact printing process, significantly reducing mechanical interactions between nanowires and allowing for precise control via surface chemical interactions. Moreover, large-scale integration of diverse device structures using nanowire arrays on flexible plastic substrates has been demonstrated, controlling semiconductor channel widths from single nanowires (approximately 10 nm) to about 250 µm, comprising parallel arrays of over 1250 nanowires that provide more than 1 mA of on-current.

In contrast, the IPSLS technique bypasses alignment concerns. To further enhance stretchability, we integrated SiNWs grown via IPSLS with island protection to create discrete SiNWs FET architectures (Fig. 7g–i) [205]. SiNWs FETs fabricated on PDMS substrates demonstrated impressive conformability to soft skin, enduring significant compressive deformations without delamination or damage. These SiNWs FETs achieved remarkable performance metrics, including a high *I*_on_/*I*_off_ ratio (≈10^6^), low subthreshold slope (< 200 mV dec^−1^), and excellent hole mobility (≈70 cm^2^ V^−1^ s^−1^), withstanding over 1000 cycles of repetitive testing at a 20% strain and exhibiting stretchability of up to 50%. In summary, flexible SiNWs FETs lay a solid foundation for exploring and integrating advanced applications in stretchable displays, wearable electronics, and sensor technologies.

### Energy Supply and Storage Devices

The development of flexible electronic devices faces numerous challenges, among which sustainable energy supply and efficient storage are critical issues. Flexible energy devices, such as solar cells [206–212], electric generators [213–216], and battery [217–222], play a pivotal role in addressing these challenges. These devices not only provide stable and continuous power to ensure the normal operation of flexible electronics in diverse environments but also enhance the compatibility and design flexibility of the devices by seamlessly integrating with flexible circuits and sensors. Additionally, their lightweight and thin characteristics make them more portable and comfortable, particularly suitable for wearable and portable applications. This section explores the applications of SiNWs in energy supply and storage.

#### Energy Supply Devices

Flexible energy supply devices can harvest energy from the environment or the human body, enabling self-sufficient power generation and reducing dependence on external energy sources. Compared to conventional silicon thin films, which face trade-offs between light absorption and carrier collection due to low carrier diffusion lengths and structural defects, SiNWs offer distinct advantages. Their high aspect ratio and radial structure effectively capture and guide incident light, minimizing reflection losses and achieving higher absorption within thinner material layers [145, 223]. Furthermore, the 3D free-standing radial junction (RJ) structure spatially orthogonalizes light absorption and carrier collection directions, significantly enhancing carrier collection efficiency [224]. This design also reduces material consumption, improves light trapping, and mitigates light-induced degradation, such as the Staebler–Wronski effect in amorphous silicon (a-Si:H) cells [225, 226]. Additionally, the RJ structure improves cell flexibility, making SiNWs a promising pathway for developing high-performance, flexible solar cells [211, 212, 224].

**Fig. 8:**
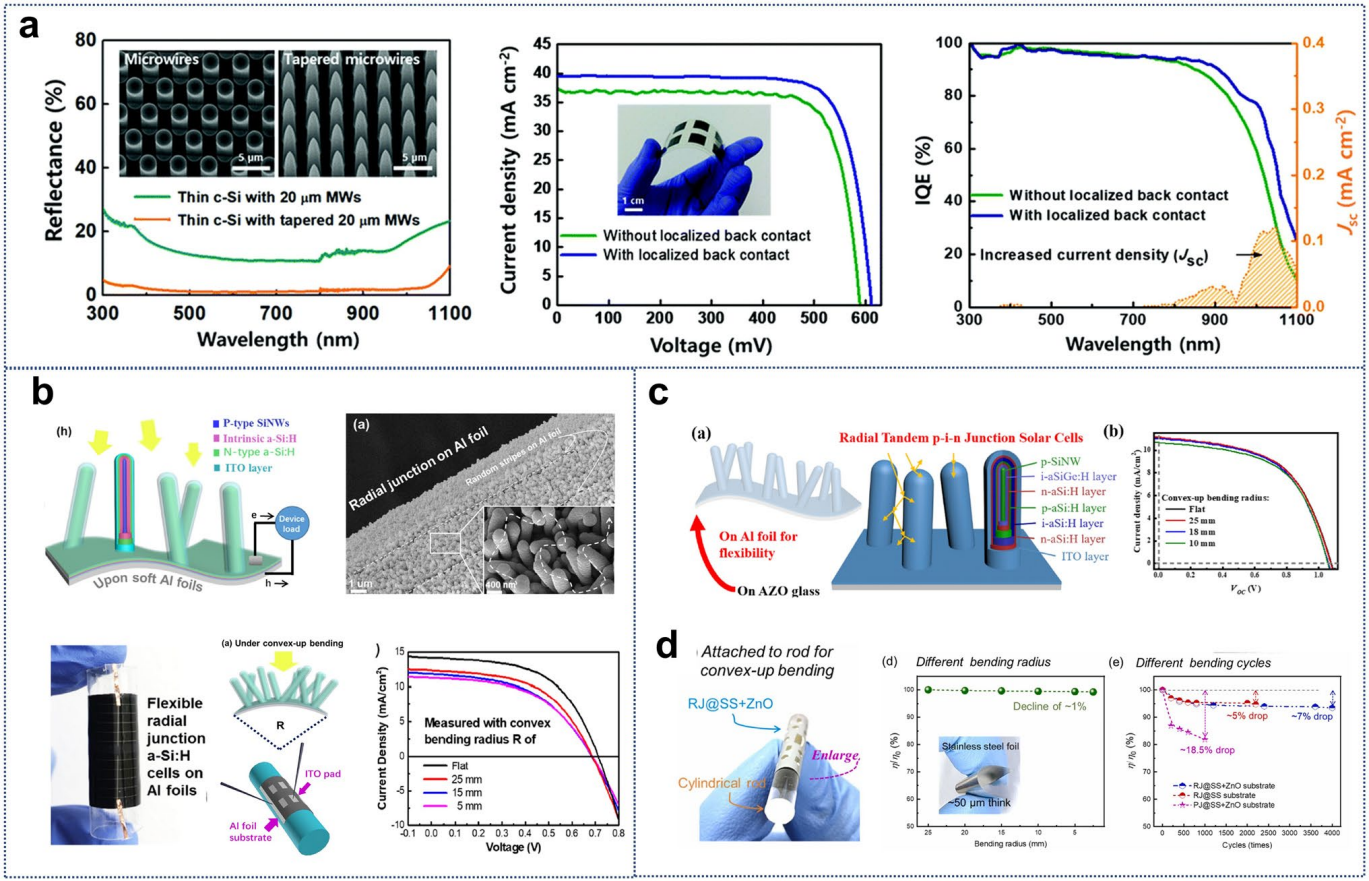
**a** Flexible c-Si RJ solar cells with vertically aligned SiNWs on c-Si through an etching process, exhibiting a constant efficiency during up to 1000 bending tests. Reproduced with permission from Ref. [227]. Copyright 2018, Royal Society of Chemistry. **b** Structure of 3D RJ solar cell on soft supermarket-available AF of 15 μm thick, and the convex-up testing J–V characteristics with different bending radii of 25, 15 mm and 5 mm. Reproduced with permission from Ref. [210]. Copyright 2018, Elsevier. **c** Schematic diagram of flexible RJ thin film solar cells fabricated on Al foil and their J–V curves vs different bending radius. Reproduced with permission from Ref. [211]. Copyright 2021, Elsevier.** d** Flexible and durable RJ solar cells fabricated on ZnO coated stainless steel substrates and their relative efficiency as a function of different bending radii and bending cycles with a radius of 2.5 mm. Reproduced with permission from Ref. [212]. Copyright 2024, Elsevier

Hwang et al. fabricated flexible c-Si RJ solar cells with vertically aligned SiNWs on c-Si through an etching process (Fig. 8a), achieving a maximum power conversion efficiency of 18.9% [227]. These SiNW-based photovoltaic cells exhibited a significant increase in current density and maintained stable efficiency even after 1000 bending cycles. Compared to the etching method, VLS-based SiNW RJ photovoltaic cells offer a more cost-effective and efficient solution, though their reliance on high-temperature (> 600 °C) Au-catalyzed growth processes is incompatible with soft substrates. Sun et al. demonstrated a robust 3D architecture for a-Si:H radial p-i-n junction solar cells on soft supermarket-available aluminum foils (AF, 15 µm thick) at < 350 °C using low-melting-point tin as the catalyst (Fig. 8b) [210]. The discrete, firmly rooted SiNWs cores on the soft AF surface provided structural support, protecting the protrusive photoactive RJ from instability in the a-Si/Al bottom layer. This structure achieved excellent flexibility and integrity, enduring 300 convex-up and convex-down bending cycles with a small radius of 5 mm.

By marrying advanced tandem junction design to the 3D SiNWs framework, Zhang et al. have successfully demonstrated high-performance 3D RJ solar cells with ultrathin absorber layers (a-Si:H: ~ 55 nm, a-SiGe:H: ~ 45 nm), as shown in Fig. 8c [211]. These RJ cells on aluminum foil sustained bending radii as small as 10 mm, achieving an 8.1% efficiency and a open-circuit voltage (VOC) of 1.2 V. Building on this capability, Wang et al. transitioned to ultrathin stainless steel foil substrates with superior mechanical strength, flexibility, and durability (Fig. 8d) [212]. A thin ZnO layer precoated on the SS surface optimized RJ density, enhancing photoelectric performance. A single-pump process produced highly flexible RJs on 50 µm SS substrates, achieving record efficiency (6.01%), VOC: 0.80 V, and short-circuit current density (Jsc: 12.87 mA cm^−2^). The 3D RJ structure demonstrated remarkable durability, with only a 7% efficiency reduction after 4000 bending cycles at a 2.5 mm radius.

**Fig. 9:**
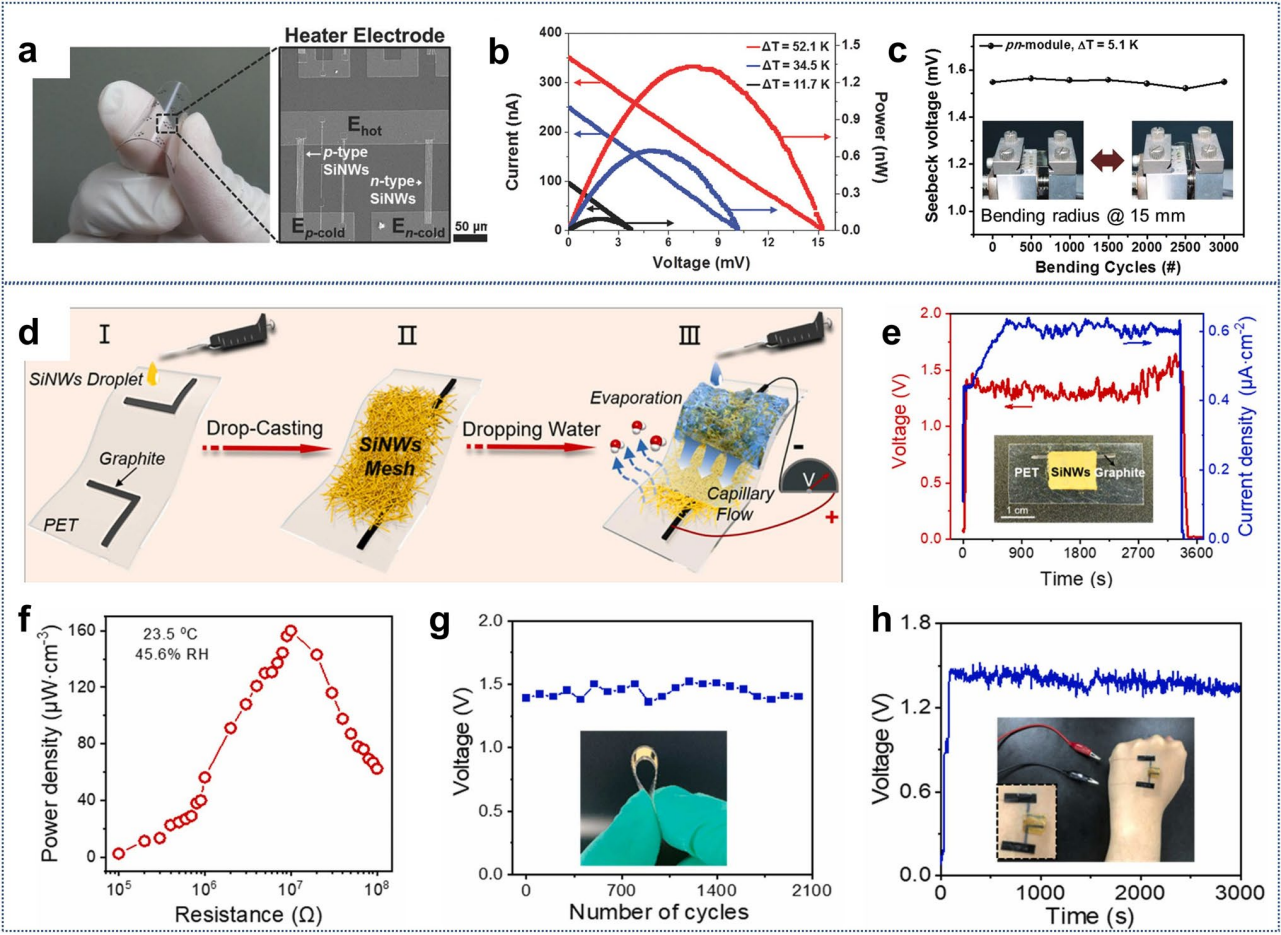
**a** Flexible thermoelectric pn-module on a plastic substrate. **b** *I–V* and power curves at temperature differences of 11.7, 34.5, and 52.1 K. **c** Seebeck voltage stability over 5000 bending cycles at Δ*T* = 5.1 K (insets: flat and convex device states). Reproduced with permission from Ref. [214]. Copyright 2016, John Wiley and Sons. **d** Fabrication and performance of SiNWs mesh-based EIEG: schematic of the drop-casting process on PET (I–III), **e** electrical output with DI water infiltration (inset: device photo), **f** power density variation with external load, **g** voltage stability after repeated bending, and **h** device output on a human hand. Reproduced with permission from Ref. [213]. Copyright 2022, Elsevier

In addition to sunlight-based photovoltaic devices, flexible energy solutions have explored other mechanisms for energy harvesting. Choi et al. reported a flexible thermoelectric generator comprising SiNWs fabricated using a top–down approach [214]. These generators utilized thermal energy, achieving Seebeck coefficients of 156.4 and −146.1 µV K⁻^1^ for p-type and n-type SiNWs, respectively. The pn module achieved a maximum power factor of 14.2 mW (m K^2^)⁻^1^, maintaining stable performance even after 3000 bending cycles (Fig. 9a–c). Additionally, Shao et al. proposed an Evaporation-Induced Generator (EIEG) based on SiNW meshes [213]. Utilizing capillary flow in SiNW nanopores, the EIEG harvested electrical energy from natural water evaporation, delivering a high open-circuit voltage (V_oc_: ~ 1.5 V) and a power density of over 160 μW cm⁻^3^ for 3500 s, with excellent mechanical durability after 2000 bending cycles (Fig. 9d–h).

#### Energy Storage Devices

While energy supply devices provide green energy solutions, efficient energy storage devices are essential for continuous power delivery. One of another challenges in flexible electronics is the development of storage systems capable of adapting to various deformations. Si has emerged as a promising candidate for next-generation lithium-ion battery electrodes due to its superior theoretical gravimetric capacity (4200 mAh g⁻^1^), significantly outperforming traditional graphite [228, 229]. However, practical application is hindered by silicon’s substantial volume expansion during lithiation, which leads to mechanical failure and capacity fading. SiNWs offer a compelling solution by mitigating these issues. Their high surface area enhances mass loading, while their nanostructured geometry helps maintain contact with current collectors during cycling, addressing both mechanical and electrochemical challenges [218–222, 228–230].

**Fig. 10:**
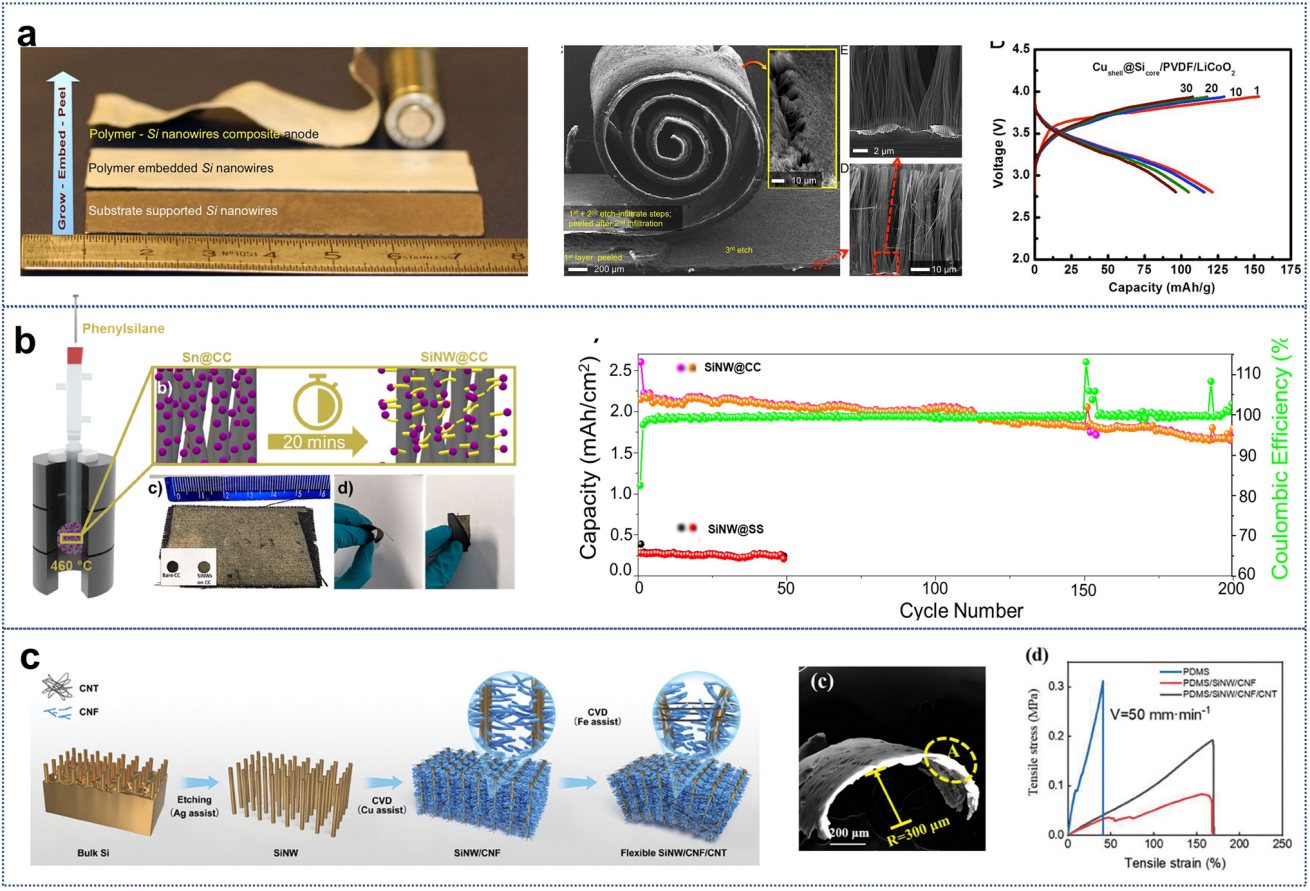
**a** Ensemble view of the large-scale LIPOSIL processing flow. Commercial battery-scale fabrication is achieved, demonstrating scalability and deformation tolerance. Reproduced with permission from Ref. [221]. Copyright 2012, National Academy of Sciences. **b** Schematic of SiNW synthesis on high-surface-area carbon cloth (CC) via a glassware-based CVD method, showing uniform SiNW coverage (matt yellow) on CC (black). Flexible SiNW@CC substrates exhibit stable cycling performance with high Coulombic efficiency and robust areal capacities under bending conditions. Reproduced with permission from Ref. [218]. Copyright 2023, John Wiley and Sons. **c** Flowchart of the synthesis of flexible SiNW/CNF/CNT arrays. CNFs act as strong joints connecting SiNWs and CNTs, while CNTs serve as the primary conductive units in the carbon chain, enabling high-loading flexible anodes. SEM image of the SiNW/CNF/CNT structure after 500 bending cycles. Stress–strain curves of PDMS and its composites with SiNW/CNF and SiNW/CNF/CNT demonstrate enhanced mechanical properties. Reproduced with permission from Ref. [222]. Copyright 2022, Elsevier. (Color figure online)

To address these issues, Vlad et al. have developed a scalable method to fabricate mechanically robust, freestanding SiNW-based membranes using an etch-infiltrate-peel cycle (Fig. 10a) [221]. Vertically aligned SiNWs, etched from recycled Si wafers, were impregnated with a polymer matrix that serves as both a lithium ion gel-electrolyte and electrode separator. A conformal porous copper nanoshell was subsequently deposited around the SiNWs, stabilizing the electrodes and enhancing current collection efficiency. This innovative design mitigated capacity loss due to volume expansion while enabling the production of mechanically flexible 3.4 V lithium-polymer silicon nanowire batteries with high aspect ratios (> 100) and scalable dimensions. The resulting devices demonstrated superior electrochemical performance, including excellent structural durability under deformation.

Building on this foundation, Storan et al. demonstrated SiNW synthesis on high-surface-area carbon cloth substrates using a VLS method at 460 °C. The flexible carbon cloth provided a stable current collector, retaining structural integrity under bending and twisting (Fig. 10b) [218]. This configuration achieved an areal capacity of 2.0 mAh cm^−2^, with porous SiNW structures maintaining 80% capacity retention after 200 cycles. Su et al. further advanced the field by integrating SiNW arrays with a carbon chain composed of short carbon nanofibers and long carbon nanotubes (CNTs) (Fig. 10c) [222]. This composite structure enhanced electrode flexibility and conductivity, while the CNT network provided mechanical stability. The SiNW-based anodes achieved an impressive initial capacity of 2856 mAh g⁻^1^, retaining 60% of their capacity (1602 mAh g⁻^1^) after 1000 cycles. Notably, the system exhibited exceptional mechanical durability, with capacity degradation of less than 1% after 100 bending cycles, highlighting its potential for high-load flexible storage systems.

### Flexible Biosensors

Flexible biosensors have emerged as a transformative technology in modern healthcare and biomedical research, leveraging the unique properties of flexible materials to enable applications in wearable devices, real-time health monitoring, and precision diagnostics. By integrating advanced materials like SiNWs with flexible substrates, these sensors achieve a remarkable balance between mechanical adaptability and high-performance sensing capabilities. SiNWs, with their nanoscale dimensions, exceptional piezoresistance, and high surface-to-volume ratio, serve as versatile building blocks for a wide range of flexible biosensors, including strain sensors, DNA biosensors, cellular detection platforms, and others.

The integration of SiNWs into flexible biosensors not only enhances sensitivity and miniaturization but also addresses challenges related to device stretchability, mechanical durability, and compatibility with dynamic biological systems. This chapter explores the advancements in SiNW-based flexible biosensors, focusing on their applications in strain sensing, molecular diagnostics, and cellular-level investigations. These developments underscore the potential of SiNWs to bridge the gap between rigid, high-performance electronic platforms and the soft, dynamic environments of biological systems, paving the way for next-generation biomedical technologies.

#### Strain Sensors

Recently, flexible strain sensors have been attracted intense attention in wearable device applications, which are applied to convert the human body’s motions into electrical signals and thus to enable precise feedback and control in applications such as human–machine interactions, healthcare and remote surgery, robotic systems. The piezo resistance effect of bulk single-crystal Si has first been discovered in 1954 [231], which can be explained by the splitting of light- and heavy-hole bands in the center of the Brillouin’s zone. Elastic strain can also change the shape of isoenergetic surfaces in these bands. Therefore, many efforts have been devoted to develop highly-sensitive strain sensors from Si active materials, due to their large gauge factor, stable mechanical properties and well-developed modern Si-based electronics platform. However, the applications of Si in flexible strain sensors have been hindered by the large rigidity and brittleness of commonly used Si wafers. Stretchable Si materials can be obtained through structural design, such as wave structures, serpentine structures, cracks, and origami. These structures can transform stretchability to the bending of thin structures. Based on these design concepts, various flexible Si materials have been designed, fabricated, and used for flexible sensors. Alternatively, more dimensions of Si materials can be reduced for better flexibility. For example, SiNWs, as a typical 1D Si nanostructure, have nanoscale diameters and 1D conductive channels. The SiNWs possess an unusually large piezoresistance effect compared with bulk. For example, the longitudinal piezoresistance coefficient along the < 111 > direction increases with decreasing diameter for p-type SiNWs, reaching as high as −3550 × 10^11^ Pa^−1^, in comparison with a bulk value of −94 × 10^11^ Pa^−1^. This giant piezoresistance effect in SiNW is induced by carrier depletion within the NWs due to mechanical stress and may have significant implications in nanowire-based flexible electronics, as well as in nanoelectromechanical systems [232].

**Fig. 11:**
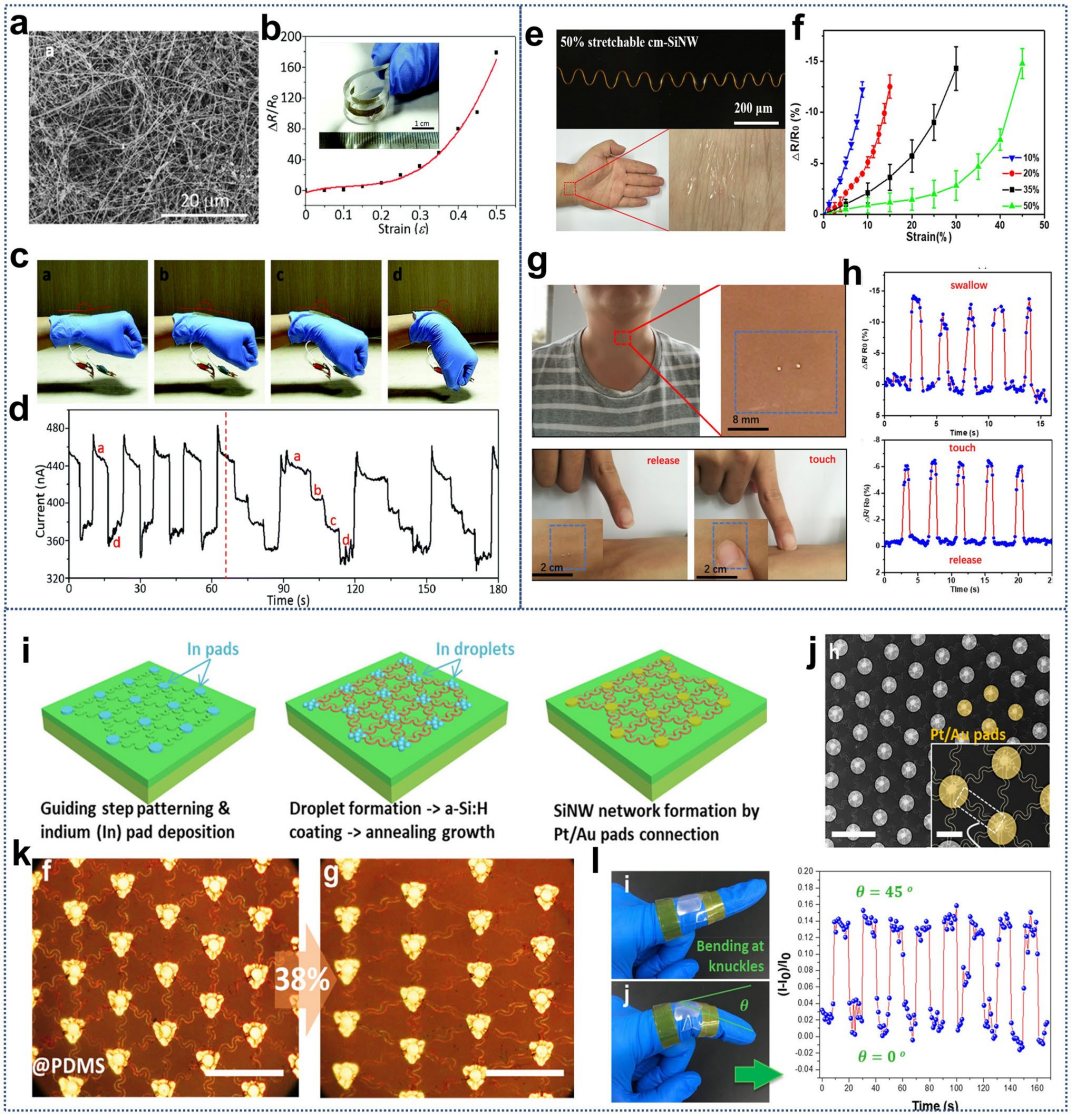
**a, b** Si fabric consisting of long Si nanowires synthesized by CVD, the insert shows the photo of the fabricated sensor embedded in PDMS. **c, d** Plot of the relative change in resistance versus strain. The digital images and current response of flexible Si fabric strain sensor to the wrist motion. **a–d** Reproduced with permission from Ref. [233]. Copyright 2009, Royal Society of Chemistry. **e** Miniaturized stretchable SiNW strain sensors (~ 50%) for E-skins applications. **f** Relationships between the ΔR/R_0_ and applied strain of strain sensors with different prestrains. **g, h** SiNW strain sensor are placed on the throat for swallow and touch motion sensing. **e–h** Reproduced with permission from Ref. [176]. Copyright 2020, American Chemical Society. **i–k** The flexible SiNW network consisting of the IPSLS spring SiNWs and the metal joins. **l** SiNW networks were mounted on a finger knuckle for the bend testing, while the current variations were recorded at different bending statuses. **i–l** Reproduced with permission from Ref. [234]. Copyright 2019, American Chemical Society

Numerous studies have also shown the potential of SiNWs to serve as miniaturized blocks for flexible electronics and highly sensitive sensors. When external stress conditions change, the electrical parameters of SiNWs, such as conductivity, resistance, or capacitance, also change accordingly. By detecting these changes, the sensor can sense and quantify alterations in the external environment. For example, Si-based flexible strain sensors are fabricated with Si fabric consisting of long Si nanowires which were synthesized by thermally evaporating a mixture powder of SiO_2_ and Sn in a tube furnace with a tube (Fig. 11a–d). The SiNWs were dispersed in ethanol to form 3 mg mL^−1^ stock solution, then the stock solution was vacuum filtered through filter paper. Finally, the fabric could be easily peeled off from the filter paper after drying [233]. The Si fabric sensors demonstrate a large strain range of 50%, high sensitivity GF of up to 350, and stable responses during 500 repeated stretch/release cycles, which are superior to the performance of the conventional Si-based rigid strain sensor.

The GF reflects the sensitivity of strain sensors and can be calculated according to the following equation:

1 $$\text{GF} = \frac{\Delta R / R_0}{\varepsilon}$$

where *R*_0_ is the original resistance, Δ*R* is the resistance variation, and *ε* is the strain.

For the high demands of miniaturized sensor devices, they further reported the novel ultraminiaturized stretchable strain sensors based on single ultralong SiNWs [176]. A single wavy centimeter-long SiNW was first transferred to a pre-strained PDMS substrate with a thin partially cured PDMS layer (Fig. 11e–h). Then, the used pre-strain was released after complete cure of the thin layer. The stretchable SiNWs with different wavy shapes can be determined by applying different pre-strains on PDMS substrates. After that, the two ends of the cm-SiNW were contacted with indium gallium alloy and copper wires to maintain a good electrical contact while stretching. Finally, the cm-SiNW and the electrodes were encapsulated with a thin PDMS layer to obtain an ultraminiaturized stretchable strain sensor. The strain sensor with 50% pre-strain could achieve a maximum strain sensing range of 45%. Durability tests involving 10,000 loading cycles at 10% strain confirmed the long-term durability of the SiNW sensors, which also withstood over 1000 cycles at 40% strain. Additionally, the sensors were not only capable of monitoring large human motions (such as wrist, elbow, and finger joint movements) but also small human movements (such as swallowing, frowning, and smiling), taking the swallowing signal detection as an example shown in Fig. 8e–h. Therefore, the sensors can serve as ultraminiaturized sensing components in imperceptible e-skins.

Although the SiNWs grown via the VLS method can be bent through strain, the scalable mass production, stable integration, and higher levels of assembly of ordered SiNW springs even large-area stretchable networks are still a challenge. Based on the high shape-designed capacity, we demonstrate the grow-in-place integration of SiNW springs into highly stretchable, transparent, and quasicontinuous functional networks, via a readily scalable, reliable, and low-temperature batch-manufacturing approach (Fig. 11i–l) [234]. The ultralong sinusoidal spring SiNWs, led by the in droplets, grew out of the in pads and followed the guiding step edges via IPSLS growth mode. We applied a unique double-lane and double-step channel design to guarantee a highly guided growth rate and thus a high-quality network interconnection and formation. The SiNW network presents a regular hexagonal layout, with a lattice spacing of *L*_hex_ = 136 μm. To join the SiNWs lying in the discrete spring channels into a continuous network, a platinum/Au electrode pads were defined by lithography and deposited by electron beam evaporation to cover the in pads and the ends of the guiding channels. The continuous SiNW network can be reliably transferred to a soft elastomer substrate to construct a mechanical sensor, which can conformally attach to highly curved surfaces and demonstrate an excellent stretchability of > 40%. The stepwise stretching-current measurements showed that the transport current increases by 17% while being stretched to ∼38%, and this variation can be well recoverable. Repetitive stretch release testing for 50 cycles (@37%) indicated that a reliable pulling strain sensing capability with a gauge factor of GF = 82.3 was achieved. Furthermore, the bending testing carried out by mounting a piece of the SiNW network sample to the knuckle of a finger also reveals a robust and repeatable bending strain response, where the transport current increases by 15% at a bending angle of 45°. Thus, this continuous SiNW network has a unique potential to eventually establish a new generically purposed wafer-like platform for constructing soft strain sensors with Si-based hard performances.

#### DNA Biosensors

The application of SiNWs in flexible biosensors represents a significant advancement in the realm of biomedical diagnostics. The unique properties of SiNWs, including their nanoscale dimensions and high surface area, enable the development of highly sensitive biosensing platforms that can be integrated into wearable technologies for real-time health monitoring.

**Fig. 12:**
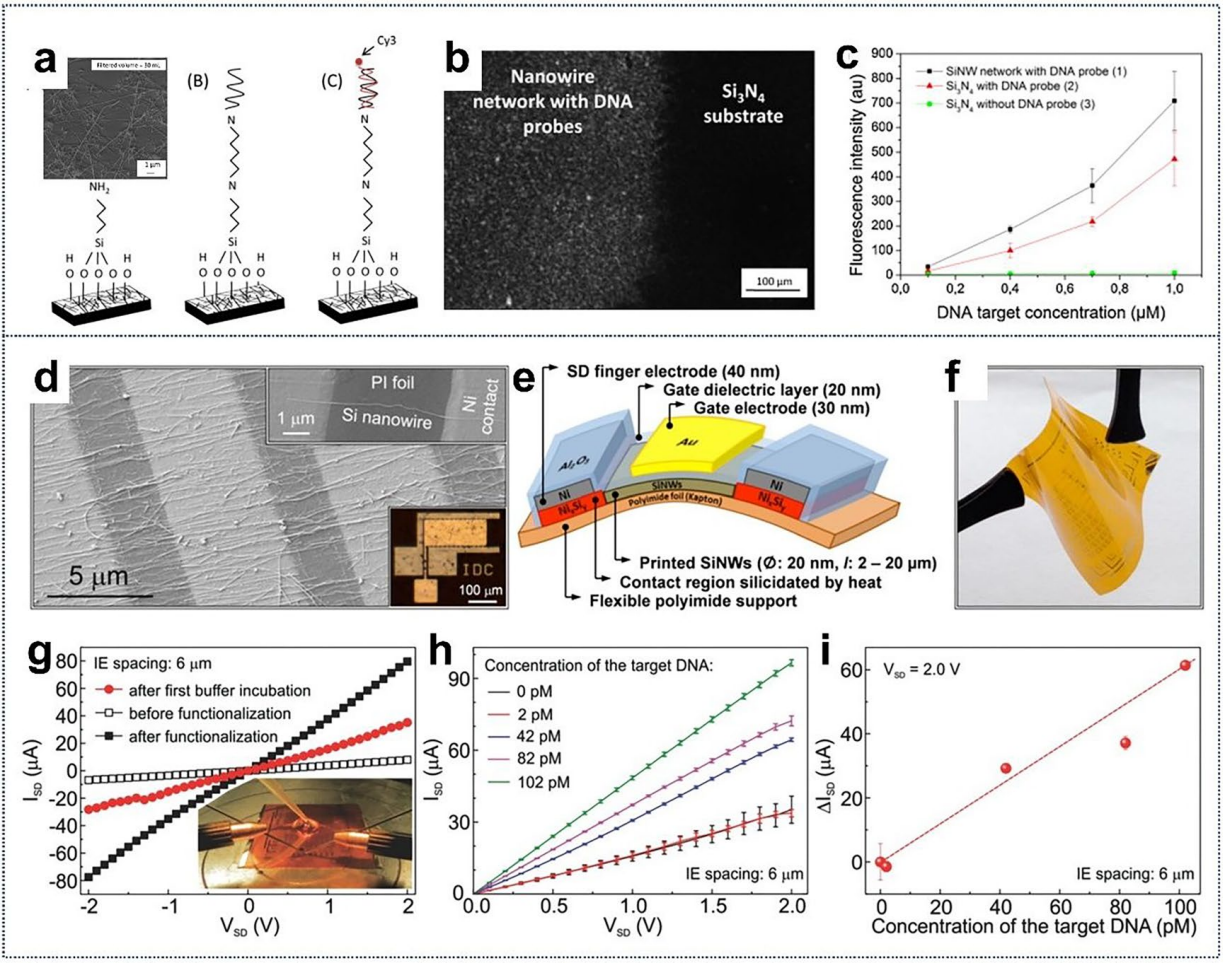
**a** Schematic of APTES modification, DNA probe grafting, and hybridization with Cy3-labeled target.** b** Fluorescence micrograph of SiNW network at 1 μM DNA target concentration. **c** Fluorescence intensity versus DNA target concentration for three areas. **a–c** Reproduced with permission from Ref. [235]. Copyright 2013, Elsevier. Lightweight, flexible SiNW-FET biosensing platform. **d** SEM image of SiNW array bridging finger electrodes. **e** Schematic cross-section of functional device layers. **f** Optical image of the diagnostic platform with bendable transistor arrays for Avian Influenza Virus H1N1 DNA detection. **g** Output characteristics of SiNW-FETs before and after functionalization. **h** Output characteristics after hybridized target DNA detection in the picomolar range (IE spacing: 6 μm). **i** Source-drain current change at VSD = 2 V versus DNA target concentration. **d–i** Reproduced with permission from Ref. [236]. Copyright 2015, John Wiley and Sons

Recent studies have explored the fabrication of silicon nanowire-based DNA biosensors, utilizing a filtration method to create reproducible and homogeneous random networks. These networks can be deposited on various substrates—rigid or flexible, conductive or insulating, transparent or opaque—at room temperature, facilitating large-scale applications. The immobilization of DNA onto these SiNW networks has demonstrated promising capabilities for detecting DNA hybridization, showcasing enhanced sensitivity and selectivity through fluorescence studies (Fig. 12a–c) [235].

Moreover, a flexible diagnostic platform has been developed specifically for the early detection of avian influenza virus subtype H1N1 DNA sequences. This platform employs SiNW FETs fabricated on flexible polyimide foils, resulting in devices that are significantly lighter and more cost-effective than their rigid Si wafer counterparts (Fig. 12d–i). [236] Notably, these devices retain their performance integrity even after extensive mechanical stress, enduring bends as tight as 7.5 mm and maintaining functionality after 1000 consecutive bending cycles. The diagnostic platform excels in analytical performance, achieving a limit of detection of 40 × 10^−12^ m within just 30 min, underscoring its potential for rapid and reliable early-stage disease diagnosis. This innovative approach not only enhances the feasibility of deploying biosensors in real-world settings but also paves the way for advanced applications in personalized healthcare, where continuous monitoring and swift diagnosis are critical.

As we delve further into the applications of SiNWs, it is evident that their integration into flexible biosensors heralds a new era in medical diagnostics, combining mechanical robustness with high sensitivity to address pressing healthcare challenges.

#### Cellular Detection

The detection of small molecular in cells, including reactive oxygen species and calcium ions (Ca^2+^), play a vital role on the understandings of life activities, causes, development and treatment of the related diseases. Currently, many fluorescent probes have been developed for selective signaling of these molecular and they possess great merits, such as high sensitivity, fast response time, [237–241]. Considering the significant differences among individuals of the same type of cells, the accurate determination of these signals at single-cell level could be used to investigate their dynamic activity and reveal the biological role. However, most fluorescent sensors only be able to investigate large numbers of cells, which just measure the average response of cell populations. Recently, freestanding nanoprobes for single-cell study at the nanoscale has been presented in forms, such as pillars, tubes, and wires. They are gently inserted into a cell and show great advantages such as active insertion into the cell at the location as we need, realizing subcellular resolution and decreasing diffusion distance, etc.

Among them, SiNWs are a competitive substrate carrier to construct a single nanowire-based sensor for biological detection in a single cell, due to their nontoxicity, high stability, easy modification, and praiseworthy biocompatibility. Particularly, it can be easily fabricated in good straightness, rigidity, and different lengths.

Recently, single SiNW-based fluorescent sensor anchored by proper molecule has been constructed for hypochlorite and Ca^2+^ detection and realized its application in an individual cell with the assistance of micromanipulation.

**Fig. 13:**
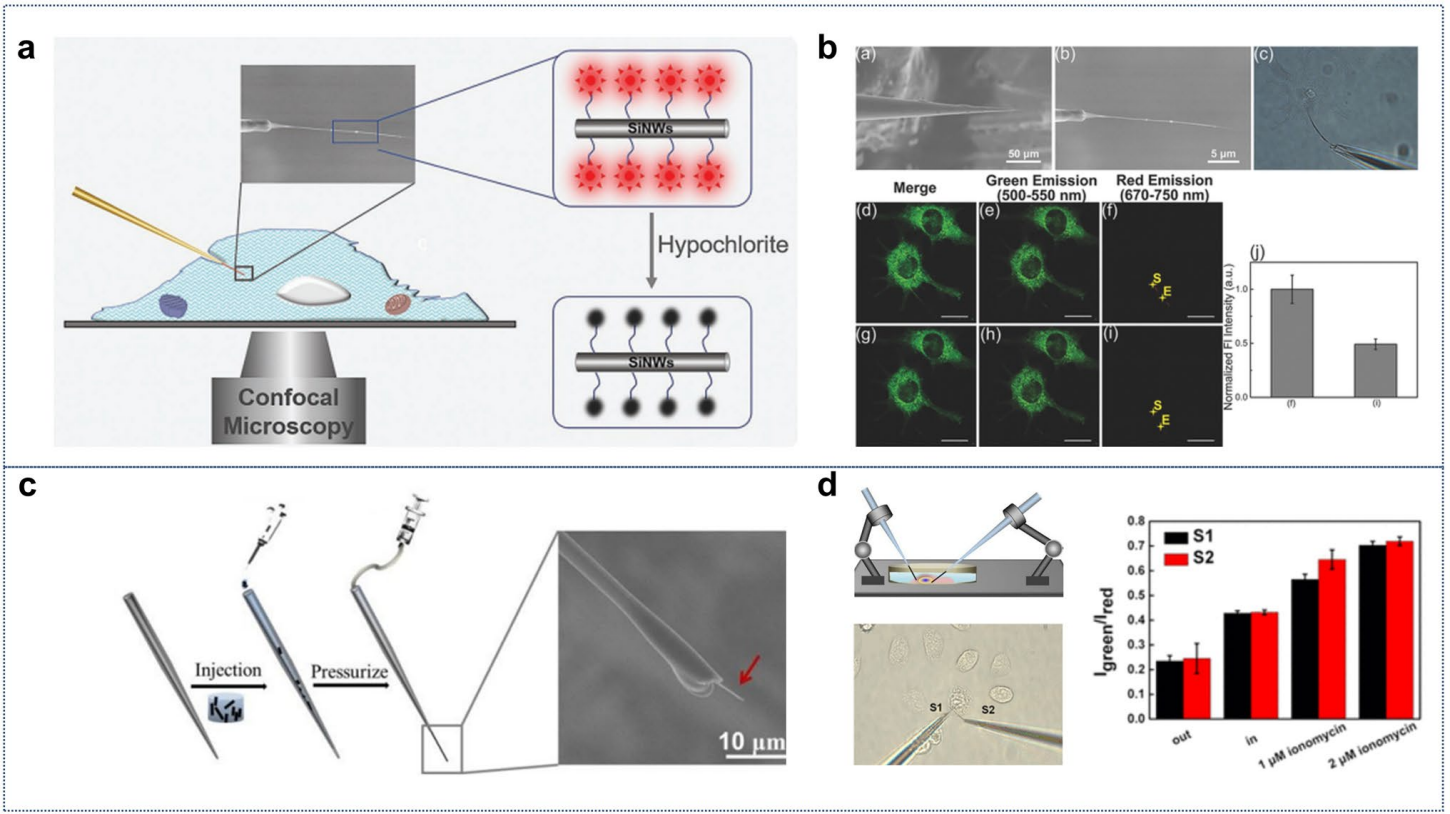
**a** Near infrared fluorescent dye decorated single SiNW-based fluorescent sensor for detection of hypochlorite in a single cell level. **b** Single SiNW sensor loaded to the tip of micropipette and inserted into a RAW264.7 cell for the detection of intracellular hypochlorite concentrations. **a, b** Reproduced with permission from Ref. [242]. Copyright 2018, John Wiley and Sons. **c** Fabrication procedure of SiNW sensor on micropipette. **d** Schematic of the detection system for the single nanowire-based ratiometric biosensor for Ca^2+^ in a single cell with two positions. The *I*_green_/*I*_red_ of the two positions had almost no difference, indicating that the sensors have the capability to detect the Ca^2+^ in a living single cell simultaneously. **c, d** Reproduced with permission from Ref. [243]. Copyright 2020, American Chemical Society

The SiNWs prepared by chemical vapor deposition were first decorated by fluorescent molecule of near-infrared dye IR780 then dispersed in ethanol solution (Fig. 13a, b) [242]. After that, the SiNW sensor suspension was injected into the micropipette and then a syringe was used to put some pressure on the micropipette until suitable length of a single SiNW sensor was revealed at the tip. Finally, the micropipette was fixed by epoxy and installed to the micromanipulator for further insertion into a selected HeLa cell. The sensors exhibited high sensitivity and linear dependence of the fluorescence intensities on the hypochlorite concentrations within 0–50 × 10^−6^ M.

Similarly, two SiNW sensors decorated with Ca^2+^ fluorescence dyes were separately placed in the body and the neurites of an individual neuron to simultaneously detect their concentrations of Ca^2+^, with the assistance of a micromanipulator and laser scanning confocal microscope (Fig. 13c, d) [243]. Specifically, the SiNWs arrays were fabricated by chemical etching (CE), followed by oxide layer coating and hydroxylated treatment. Then, the SiNWs were decorated with 3-aminopropyltriethoxysilane, Ru(bpy)_2_(mcbpy-O-Suester) (PF_6_)_2_ and Fluo-3 and loaded in the tip of a micropipette by a pushing pressure. The SiNW sensors profiled the heterogeneity of the Ca^2+^ in an individual cell with a high spatial resolution, providing a new opportunity to investigate cellular metabolism by combining the advantages of a single-cell detection technique and physiology.

**Fig. 14:**
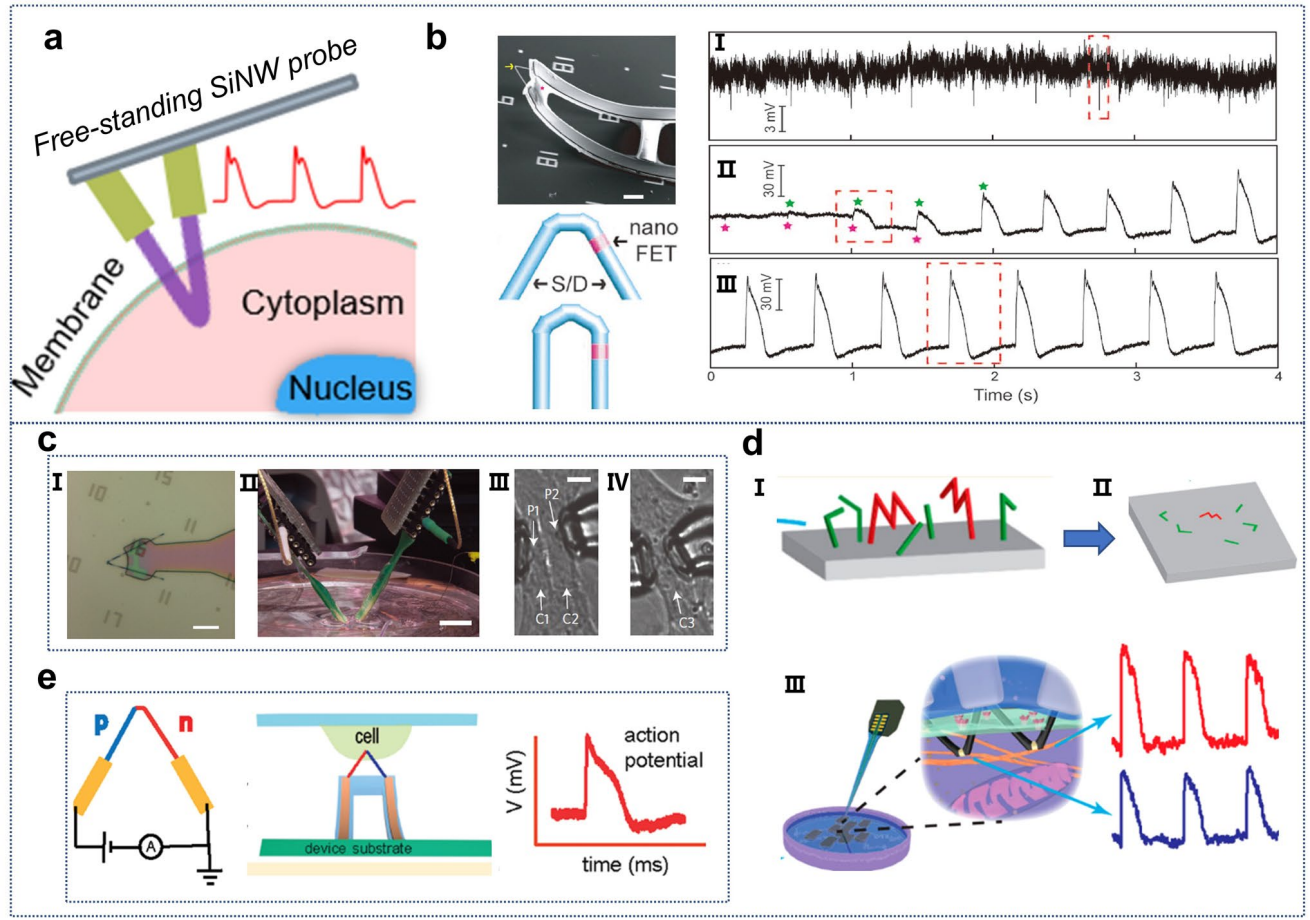
**a** Schematic of intracellular recording using free-standing SiNW probes that pierce cell membranes non-invasively. **b** The first 3D free-standing Si-FET intracellular recording probe with kinked SiNW structures: i extracellular recording, ii transition to intracellular recording during cell entry, and iii steady-state intracellular recording. Reproduced with permission from Ref. [245]. Copyright 2016, American Association for the Advancement of Science. **c** Multiplexed recording with free-standing nanowire probes: i optical image of a kinked SiNW tip on the SU8 probe body, ii setup with two independent XYZ manipulators, iii, iv multiplexed recording of adjacent cardiomyocytes (C1, C2) and submicron-spaced probes on the same cell (C3). Reproduced with permission from Ref. [62]. Copyright 2013, Springer Nature. **d** Multiplexed bioprobe fabrication: i VLS growth of kinked SiNWs, ii transfer and probe body fabrication via lithography, and iii probe release and assembly for targeted measurements. Reproduced with permission from Ref. [246]. Copyright 2014, American Chemical Society. **e** Schematic of kinked p–n junction nanowire probes for intracellular recordings from embryonic chicken cardiomyocytes on PDMS. Reproduced with permission from Ref. [170]. Copyright 2012, American Chemical Society

Recording intracellular (IC) bioelectrical signals (action potential) is central to understanding the fundamental behavior of cells and cell networks in neural and cardiac systems. The patch clamp technique, in which a glass micropipette filled with electrolyte is inserted into a cell, is considered as the standard tool for IC recording and offers both high signal-to-noise ratio and temporal resolution [244]. However, it remains limited in terms of reducing the tip size, the ability to reuse the pipette and ion exchange with the cytoplasm. Ideally, the micropipette should be as small as possible to increase the spatial resolution and reduce the invasiveness of the measurement, but the overall performance of the technique depends on the impedance of the interface between the micropipette and the cell interior. Recent efforts have been directed toward developing new advanced chip-based tools, including micro-to-nanoscale metal pillars, multiplexed nanowire transistor-based multielectrode arrays, and nanotube devices. Among them, the FET probes are considered as the ideal candidates for recording the electric potentials inside cells, because they can be made much smaller than micropipettes and microelectrodes, potentially minimizing the probe’s invasiveness. Moreover, their performance of signal detection does not depend on impedance, because they do not rely on charge transfer between the probe and the measured specimen, thus impedance-related limitations do not apply. Thus, intracellular electrical recording may be maintained for longer durations without altering the cellular integrity. For the signal detection in selected single cell, Tian et al. first demonstrated a 3D free-standing Si-FET-based intracellular recording probe with kinked silicon nanowire structures in 2010 [245]. The reproducible 120° SiNW kinks were produced by using a variation of reactant pressure during VLS process in CVD system. Connecting two 120° kinks in a cis-configuration resulted in a 60° kink. Then, a point-like localized FET were created by doping the junction region with a 3D geometry, which allows it to be introduced into cells. The electrical interconnects to the source and drain (S/D) nanowire arms were performed on ultrathin SU-8 polymer substrate. The probes bend upward by using interfacial stress that produced by mechanical mismatch between the SU-8 substrate and the metal interconnect layers. The sensing performances of the nanoscale point-like were measured on cultured embryonic chicken cardiomyocytes (Fig. 14a). With the help of phospholipid bilayer, the probe can be penetrated into the cells by internalization, without further pushing of the probe. Compared to the extracellular signals, the new peaks with opposite signs, larger amplitudes, a longer duration, and similar frequency was observed. The peaks rapidly reached a steady state (Fig. 14b) with an average calibrated peak amplitude of ~ 80 mV and duration of ~ 200 ms, which are coincident with those reported for whole-cell patch clamp recordings. However, the kink configuration and device design places limits on the probe size and the potential for multiplexing.

They further assembled these kinked SiNW probes with nanoFETs over a movable manipulator, whose position could be controlled with high spatial resolution and sub-micrometer precision (Fig. 14c). These probes can be manipulated in three dimensions within a microscope to target specific cells or cell regions, and record stable full-amplitude action potentials from spontaneously beating cardiomyocytes. They demonstrated real-time monitoring of AP changes as different ion-channel blockers are added to cells, and multiplexed recording from cells by independent manipulation of two independent nanoFET probes. This capability allows for the investigation of intercellular interaction and the electrical communication between cells with spatial precision, which is very difficult to be achieved with traditional patch clamp approach [62].

Note that, the above fabrication procedures could only fabricate single-kinked SiNW probes, which face efficiency and yield limitations when working with more complex nanoscale building blocks to integrate, especially for the multiplexed recordings or additional functionalities. Xu et al. directly construct the first multiplexed free-standing bioprobes based on w-shaped silicon kinked nanowires using gold-nanoparticle catalyzed VLS growth method with precise dopant and geometric control [246]. Specifically, two nanoscale field-effect transistors (nanoFETs) are synthetically integrated at the elbows of the silicon nanowire with three kinks in trans-orientations (Fig. 14d). The distance d between the two nanoFETs can be accurately adjusted by controlling the growth time of the nanowire arm L between adjacent kinks. Simultaneous recording of intracellular action potentials from a single spontaneously beating cardiomyocyte have been obtained by using a single probe.

Though the above studies open up a new insight for integrating electronics with cells and tissue, they are also potentially limited in that the realization of an ultimate point-like nanoFET detector is still a big challenge. In this regard, nanoscale p–n diodes are attractive since the device element is naturally localized near the junction (Fig. 14e). Therefore, Jiang et al. designed and synthetically embedded the nanoscale axial p–n junctions at the joints of kinked SiNWs. The junction can be tuned to work as a highly localized field-effect sensor to detect charges down to a single nanoparticle level and to record full intracellular signals of spontaneously beating cardiomyocyte cells [170]. Compared to the nanoFET probes, this gateable p–n diode device represents several unique advantages, such as highly localized sensing region, tuneable types of field-effect sensors and the potential use as 3D nanoscale photodetector.

As the kinked SiNWs are usually grown as random vertical bundles, they have to be broken, selectively transferred, and connected via costly and low-yield procedures, such as pick-and-place manipulation and the use of EBL. Thus, the current SiNW probes have relied on one-by-one fabrication that has been difficult to scale up.

**Fig. 15:**
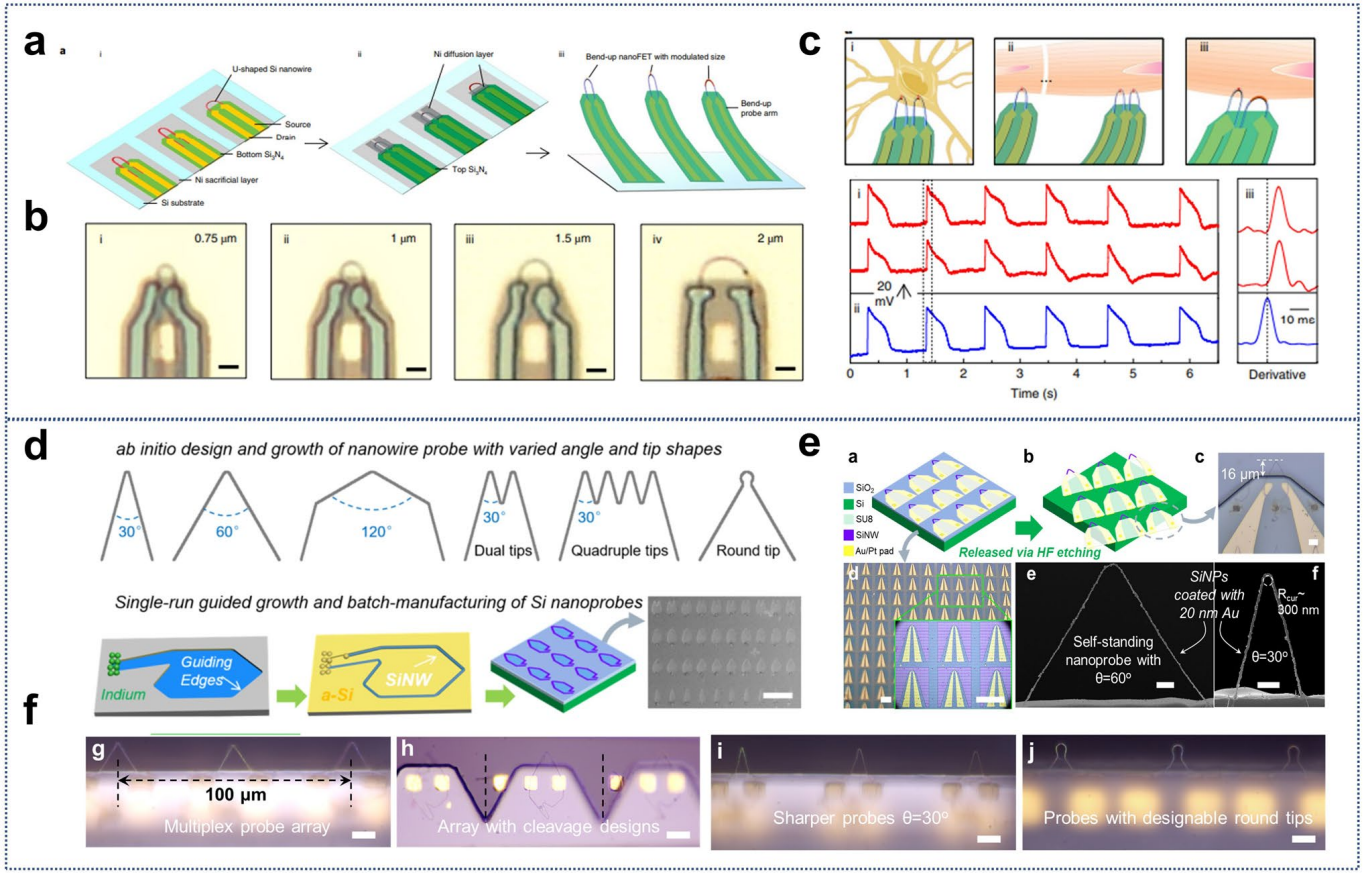
**a** Schematics of U-NWFET probe fabrication. i U-shaped nanowire assembly on a Ni sacrificial layer with Si_3_N_4_ passivation and Cr/Au/Cr interconnect deposition. ii Formation of local FETs by NiSi creation on nanowire tips after adding Si_3_N_4_ passivation and Ni diffusion layers. iii Upward bending of probes after etching Ni layers. **b** Optical images of U-NWFET probes with different radii of curvature (ROC): i 0.75 μm, ii 1 μm, iii 1.5 μm, iv 2 μm. **c** Schematics of recording scenarios: i paired U-NWFETs on one arm for multisite intracellular recording, ii multiplexed intracellular recording from different cells using separate arms, iii simultaneous intracellular/extracellular recording from one cell by paired U-NWFETs. **a–c** Reproduced with permission from Ref. [175]. Copyright 2013, Springer Nature. **d** Ab initio SiNW probes design with varied angles and tip shapes; batch fabrication via guided planar growth of SiNWs along predesigned edges. **e** Scalable assembly of free-standing SiNW probes: SU8 coating, electrical connection, and transfer of orderly SiNW probe arrays, with an optical image showing a suspended SiNW probe extending ~ 16 μm from the SU8 edge. **f** Optical images of transferred SiNP arrays with different tip angles and shapes. Reproduced with permission from Ref. [247]. Copyright 2021, American Chemical Society

To address this challenge, Zhao et al. combined their previous reported deterministic shape-controlled nanowire transfer approach with spatially defined semiconductor-to-metal alloying technology to realize scalable nanowire FET probe arrays with controllable tip geometry and sensor size [175]. The schematics of device fabrication are shown in Fig. 15a–c. The U-shaped nanowire arrays with controllable radii of curvature (ROC) were first assembled on a Ni sacrificial layer and bottom Si_3_N_4_ passivation layer by large-scale, shape-controlled deterministic assembly approach. Metal interconnects of Cr/Au/Cr to transferred U-shaped nanowires are then deposited, where the relative Cr/Au/Cr thicknesses yield a built-in strain that bends the probe up upon release. After that, the top Si_3_N_4_ passivation layer and the Ni diffusion layer were deposited, followed by rapid thermal annealing to transform the Si nanowire segments underneath and adjacent to the Ni diffusion layer to NiSi, thus generating a local FET at the tip of the U-shaped nanowire. Finally, the probe arrays will bend upward after etching the Ni diffusion and sacrificial layers. Using this unique technology, U-NWFET probe arrays with ROC from 0.75 to 2 μm and active channel lengths from 50 to 2000 nm can be batch-manufactured and used to probe cultured primary neurons and human cardiomyocytes. This high-amplitude intracellular recording capability allows for multiplexed recording from single cells and cell networks.

Although significant advances have been made in developing devices for intracellular probes, a scalable fabrication of these tiny SiNW probes with ab initio geometry designs has not been achieved. Recently, our group demonstrated a novel growth shaping approach of slim SiNWs into SiNW probes with sharp tips (curvature radii < 300 nm), with tunable angles of 30°, 60°, to 120°[247]. The growth procedure of orderly SiNW probe array via planar guided growth of SiNWs along predesigned guiding edges is illustrated schematically in Fig. 15d. The patterning of step edges by etching 150 nm deep into the underlying wafer substrate precoated SiO_2_ layer, followed by the evaporation of In catalyst pads at the ends of the guiding steps via conventional lithography, thermal evaporation, and lift-off procedure. Then, a H_2_ plasma treatment of the indium (In) pads, transforming them into discrete droplets, followed by the coating of amorphous Si (a-Si) layer as precursor. In the next step, the substrate was heated to 350 °C to activate the molten In droplets to absorb the a-Si layer to produce crystalline SiNWs. During this IPSLS growth, the extra a-Si coated on the vertical sidewalls of the step edges can help to attract and direct the movement of the leading In droplets, and thus producing SiNWs along the guiding edges. Finally, the remnant a-Si layer was selectively etched off, leaving the SiNW structure as seen in the SEM image shown in the rightmost panel.

**Fig. 16:**
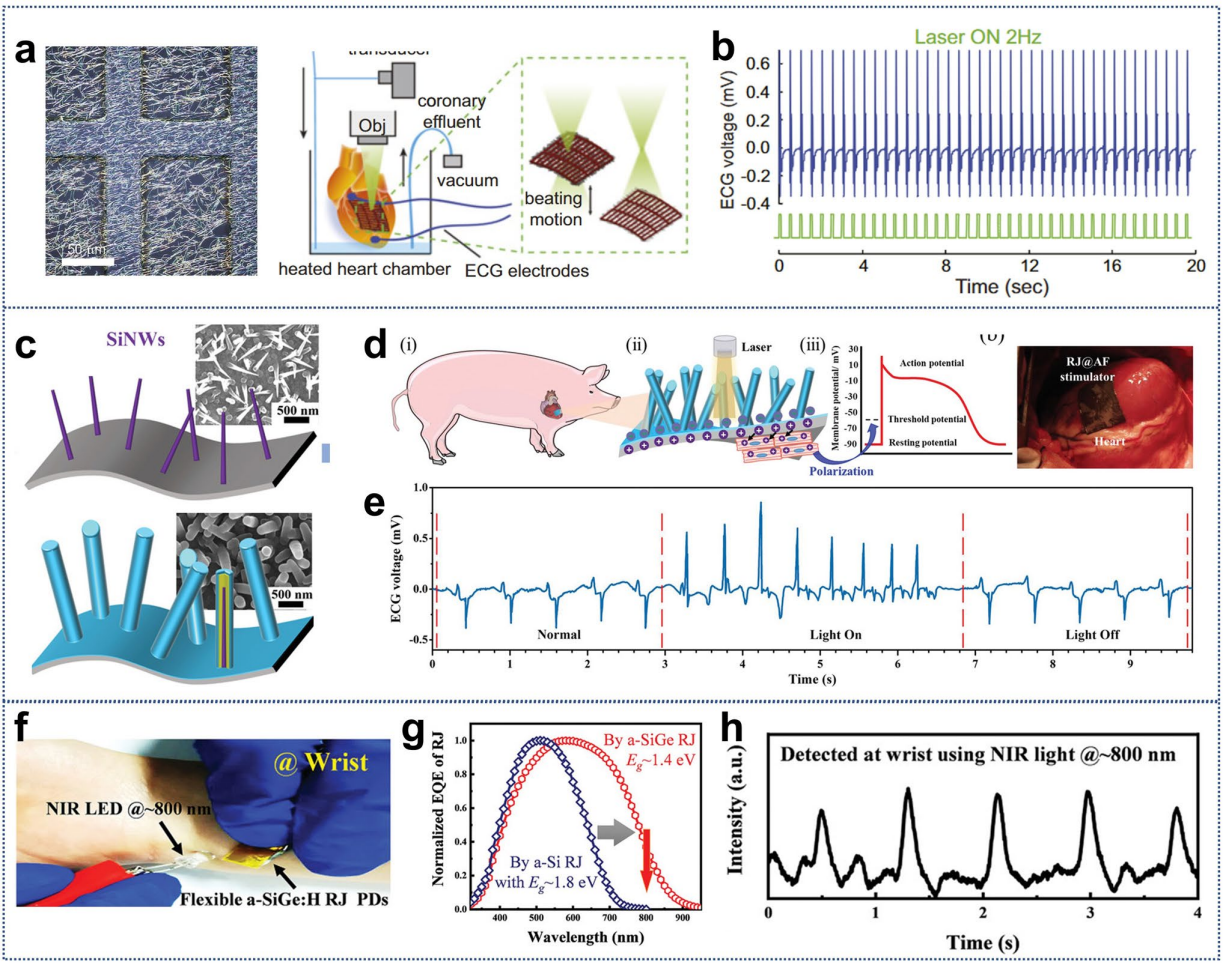
**a** Microscopy image of an SU-8/PIN-SiNW mesh composed of VLS-grown SiNWs supported by SU-8, **b** applied to the myocardium of an adult rat heart in a Langendorff setup for optical stimulation with 532 nm laser pulses at 2 Hz. **a, b** Reproduced with permission from Ref. [249]. Copyright 2019, National Academy of Sciences.** c** Flexible and degradable amorphous Si photoelectric stimulator RJ stimulators on Al substrate. **d** Photoelectrical pacemaker attached to the heart surface of living pig for cardiac pacing experiment, **e** with a photograph of the experiment configuration and recorded ECG voltage signals recorded before, under and after light stimulation. **c-e** Reproduced with permission from Ref. [63]. Copyright 2019, John Wiley and Sons. **f–h** Flexible a-SiGe RJ near-infrared photodetectors for rapid sphygmic signal monitoring at wrist. Reproduced with permission from Ref. [250]. Copyright 2021, John Wiley and Sons

For the scalable assembling of the free-standing SiNW probes, the SiNW array grown on the SiO_2_-coated wafer substrates were first connected by Pt/Au metal electrode pads, made via photolithography, evaporation, and lift-off procedure, as depicted in Fig. 15e. Then, a SU8 framework of 20 μm thick was spin-coated and patterned, covering the metal pads and holding the SiNPs at the roots. After that, the SiNW probes were released from the parent substrate, by etching off the underlying SiO_2_ layer, and transferred to movable arms (Fig. 15e). The transferred and suspended SiNW probe is presented in Fig. 15f, which extends ∼16 μm away from the SU8 holding edge. The SEM images of the free-standing SiNW probes confirm again the very well-defined slim SiNW probe profile with a rather sharp tip down to *R*_cur_ ∼ 300 nm, without the use of costly and inefficient EBL lithography. Moreover, SiNW probes of different tip geometries can be easily fabricated and assembled as a multiplex probe array, indicating a reliable and high yield procedure that can accomplish a precise growth/assembling control over huge dimension scales. The field-effect sensing functionality of the SiNW probes were systematically testified in liquid nanodroplet and cell environments. High response to tiny droplets with different pH values and easy piercing into the cells enable the SiNW probes wide range of new intracellular sensing, monitoring, and editing applications.

### Flexible Electronics for Biointerface

Flexible biosensors are highly efficient in detecting biological signals and have found extensive applications in health monitoring. However, the functionality of biosensors extends beyond mere signal detection. They can also modulate cellular and tissue behaviors through biointerface stimulation, thereby enabling broader applications in clinical therapy and biomedical engineering. Unlike traditional sensors, biointerface stimulation technologies do not solely record bioelectrical signals but rather regulate biological responses by precisely controlling external stimuli, such as modulating neuronal excitability or cardiac rhythms. Thus, building on the foundation of biosensing, advancements in flexible electronic technologies allow not only the perception of signals but also the active regulation of biological functions, driving innovative applications of bioelectronic devices in the biomedical field.

The electrical conduction system plays a crucial role on the coordinated contraction of cardiomyocytes to produce heartbeats. Abnormalities in this system can lead to delayed mechanical activation of specific regions of the heart or pathologically slow heart rates (bradyarrhythmia). Implanted electronic pacemakers are considered as the standard of care in both cardiac resynchronization therapy and bradyarrhythmia. However, the current pacemakers are usually bulky and rigid electronics that are constraint by limited battery lifetimes, and need to be installed and repaired via surgeries that risk secondary infection and injury. In addition, they require the use of physical wires that can limit the spatial resolution and location of the treatment, and can foul in the physiological environment of the chest cavity as well as produce unpredictable and unwanted electrochemical reactions at the tissue interface. Recently, optical methods for modulating cellular behavior have been developed in both fundamental and clinical applications. For example, Parameswaran et al. used a-Si radial junction (PIN) SiNWs, consisting of p-doped cores, and intrinsic and n-doped shells, to wirelessly and photoelectrochemically modulate primary rat dorsal root ganglion neuron excitability [248]. The p-type SiNW cores were first synthesized via a gold (Au) nanoparticle (NP)-catalyzed VLS growth, and then i-layer and n-layer were sequentially deposited in CVD system. On light stimulation at a neuron/PIN-SiNW interface, electrons move toward the n-type shell and holes to the p-type core, inducing a cathodic process at the n-shell that can locally depolarize a target neuron. These currents can elicit action potentials in single neurons through a primarily atomic gold-enhanced photoelectrochemical process.

Then, they further developed a freestanding flexible polymer-SiNW mesh containing a random network of the PIN-SiNWs for the optical stimulation of cultured primary cardiomyocytes as well as adult hearts ex vivo [249]. The composite mesh was fabricated using SU-8 as the polymer support component and PIN-SiNWs as the semiconductor modulation component, with mechanical transfer and subsequent photolithography. The resultant composite (SU8/SiNW) contains a high-density random PIN-SiNW network spanning across the 86 μm × 424 μm window regions in the SU8 grid. The optical stimulation for adult rat hearts ex vivo using a Langendorff setup (Fig. 16a). The epicardia of the hearts were removed in the left ventricle, and a SU8/PIN-SiNW mesh was placed onto the exposed myocardium. The flexible mesh conformably wraps around and adheres to the wet curved surface of the myocardial tissue via capillary action without the need for sutures or tissue adhesives. Figure 11a shows the electrocardiogram recordings from an adult heart with a SU-8/PIN-SiNW mesh during stimulation with 532 nm laser pulses at 2 Hz. The heat beating change from a baseline of 0.9 Hz to the targeted 2 Hz after 5 min of 532 nm pulsed light exposure (Fig. 16b). These results demonstrate an easily implemented optical stimulation method for hearts ex vivo.

Our group reported a flexible self-powered photoelectric cardiac stimulator based on hydrogenated amorphous Si (a-Si:H) radial p-i-n junctions (RJs), constructed upon standing Si nanowires grown directly on aluminum thin foils (Fig. 16c) [63]. The flexible and self-powered cardiac a-Si RJ stimulator can be conformally attached to the uneven heart surface to directly pace heart-beating under modulated 650 nm laser illumination, where the heart rate can be effectively controlled by the external photoelectric stimulations, to increase from the normal rate of 101 to 128 beating min^−1^ (Fig. 16d, e). Importantly, the a-Si:H RJ units are highly biofriendly and biodegradable, with tunable lifetimes in phosphate buffered saline environment controlled by surface ITO coating, catering to the needs of short term or lasting cardiac pacing applications. This implantable a-Si:H RJ photoelectric stimulation strategy has the potential to establish eventually a self-powered, biocompatible, and conformable cardiac pacing technology for clinical therapy.

The flexible 3D RJ structure can also be used to detect sphygmic signals which have been widely used to reflect the heat beating and can provide important information for the diagnostic of heart related disease, relying on the famous photoplethysmography (PPG) technique. The signals are extracted from the variation of light reflected/diffracted from the arterial blood volume vibration, directly caused by the heartbeats. The detectors transfer these vibration signals into electrical signals, which can be easily detected by the external connected devices. Since the near-infrared (NIR) probing light at ≈800 nm can penetrate deeper into the human skin/tissue we developed flexible hydrogenated amorphous silicon germanium (a-SiGe:H) p-i-n RJ PDs directly upon soft Al foils [250]. The a-SiGe:H thin film was used as the absorption layer, which can have a narrower band gap of *E*_g_ ≈ 1.4 eV to extend the light absorption wavelength up to > 880 nm. Compared with the traditional pulse oximetry PPG that relies on the absorption difference of oxyhemoglobin and deoxyhemoglobin to red (≈625 nm) and green (≈530 nm) probing lights, this NIR photovoltaic detector can avoid the produce of inaccurate signals of significant movement artifact or cardiac arrhythmia and exclude the influence of oxyhemoglobin/ deoxyhemoglobin absorption. The 3D a-SiGe:H RJ-PDs demonstrate excellent flexibility and mechanical stability that can undergo 1000-times bending to 10 mm radius, with a high responsivity of 140 mA W^−1^ at 800 nm and rapid rise/fall response time of 5.4/17.6 μs. The sphygmic signals at the wrist, with significant arterial blood volume changes, were successfully detected by this PPG technology, with the heart rate of 78 ± 5 beats per minute (Fig. 16f–h). These unique capabilities have a promising potential to establish a reliable and conformable PPG detection technology, which can serve as a complementary and potentially useful new diagnostic dimension for efficient and accurate real-time health monitoring.

As research into the functions of biointerfaces continues to deepen, SiNWs have emerged as a material of increasing interest due to their unique electrical properties and excellent biocompatibility. SiNWs not only facilitate efficient electrical signal conduction but also exhibit flexibility and tunability, making them ideal foundational materials for bioelectronic devices. Through careful design of the geometric shape and surface chemical properties of SiNWs, researchers are developing multi-functional bioelectronic interfaces capable of effective interaction with cells and real-time monitoring of both intracellular and extracellular electrical activities and biochemical signals.

**Fig. 17:**
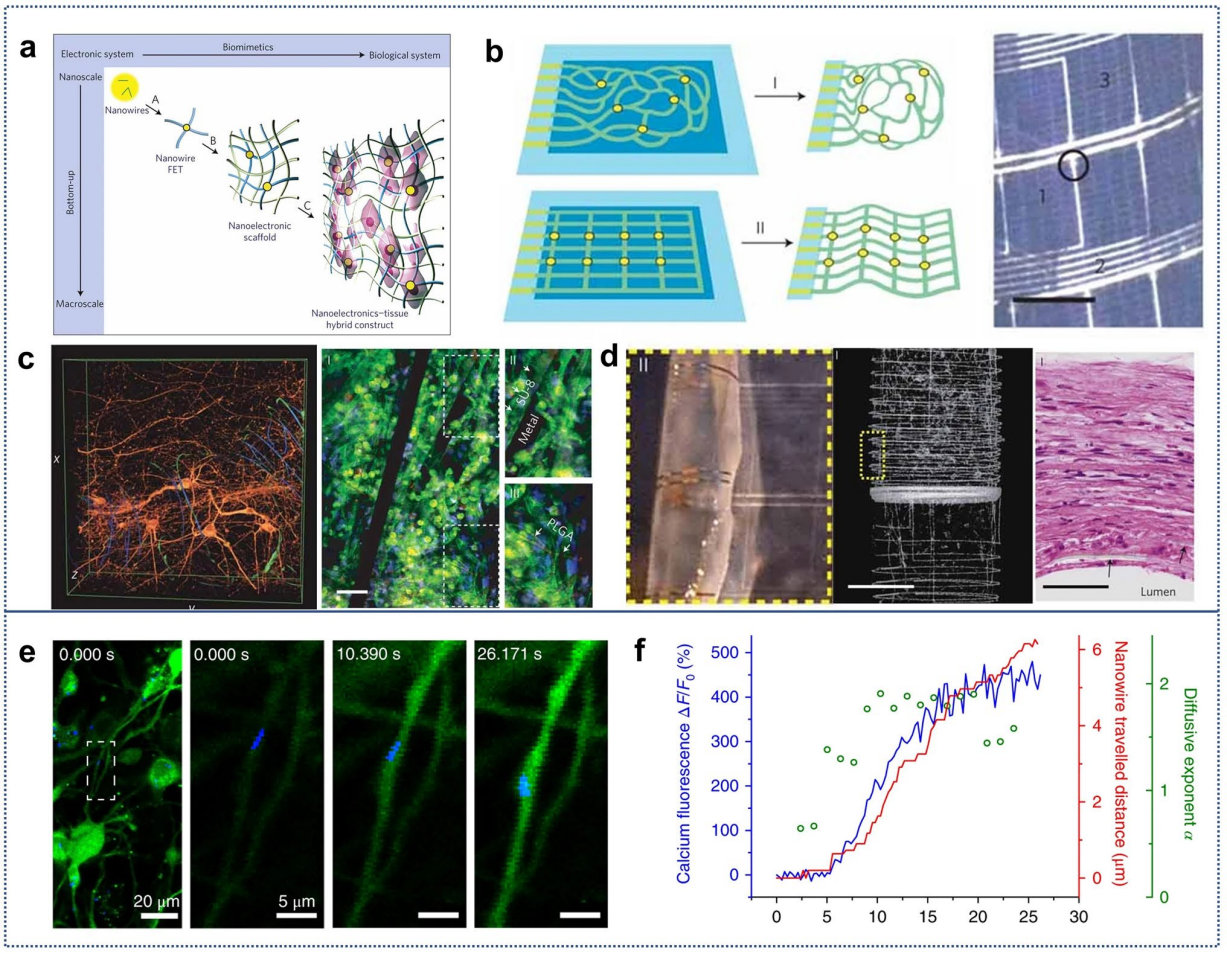
Silicon nanowire nanoelectronic scaffolds for synthetic tissue. **a** Schematic representation of the integration of nanoelectronic technology with cells and tissues. **b** Schematic illustration of the fabrication of mesh nanowire FET devices. **c** Three-dimensional cell culture within electronic scaffolds. **d** Synthetic vascular structures with sensing functionalities. Silicon Nanowire-Supported Intracellular Stimulation Interfaces. **a–d** Reproduced with permission from Ref. [251]. Copyright 2012, Springer Nature. **e** Silicon nanowires serving as dual-function intracellular biophysical tools, acting as calcium regulators and markers for motor protein-microtubule interactions. **f** Dynamic parameter variations regulating the processes. Reproduced with permission from Ref. [252]. Copyright 2018, Springer Nature

In recent years, flexible bioelectronic scaffolds have gained significant attention, with studies by Tian et al. demonstrating that SiNW-based scaffolds can mimic the microenvironment of natural tissues and possess the ability to detect electrical signals in three-dimensional space (Fig. 17a, b) [251]. This innovation not only enhances the electrical performance of biomaterials but also advances their application in the 3D culture of neurons (Fig. 17c), cardiomyocytes, and smooth muscle cells (Fig. 17d). The application of SiNWs, in conjunction with synthetic or natural biomaterials, enables precise monitoring of cellular electrical activities, assessment of drug effects, and pH variations within tissues.

Furthermore, Si-based bioelectronic interfaces provide new possibilities for non-invasive interactions between cells and electronic devices. Utilizing light-induced signal modulation techniques, these interfaces can effectively regulate intracellular calcium dynamics, cytoskeletal structures, and transport functions, thereby influencing neurotransmitter release and brain activity (Fig. 17e, f) [252]. Such research not only offers new insights into the complex interactions between biomaterials and cells but also lays the groundwork for the future development of more efficient and versatile bioelectronic devices. Consequently, the prospects for silicon nanowires in the field of biointerfaces are undoubtedly promising, heralding significant advancements in clinical therapies and biomedical engineering.

### Flexible Si Nanoelectromechanical System

The unique shape design capability of IPSLS strategy can also be used to morph SiNWs for special flexible applications, including ultracompact finger-like robotics, NW slingshots and 3D NEMS.

**Fig. 18:**
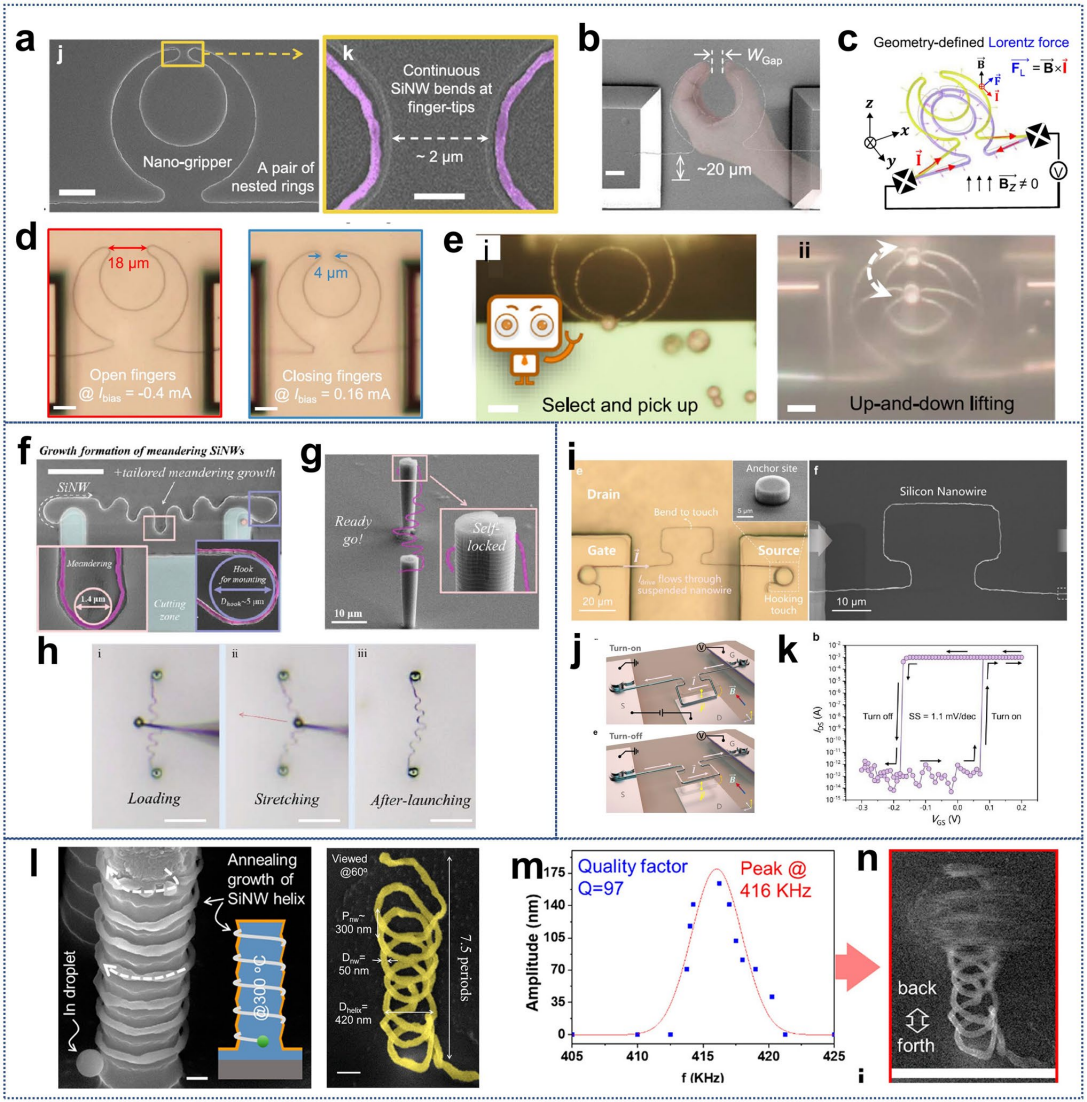
**a**, **b** Ultracompact single-nanowire-morphed grippers. A double-nested omega rings were first grown upon patterned SiO_2_/wafer substrate, and transferred onto a platform for suspending. **c** Schematic illustration of the opening and gripping deformations of a NW gripper, driven by geometry-defined Lorentz forces (FL) within a normal magnetic field in z-direction (Bz). **d** Images of the opening and closing states of the gripper, recorded at constant electric biases of *I*_bias_ = −0.4 and 0.16 mA. **e** NW gripper can select, pick up and transfer the microscale objects from substrate, and up–down lift them in z-direction. **a–e** Reproduced with permission from Ref. [64]. Copyright 2023, Springer Nature. **f, g** Meandering IPSLS SiNW can be mounted upon standing pillar frames by a unique self-locked structure design. **h** Photoshoots of a fiber tip picking up a microsphere payload, loading it to a poised NW slingshot, stretching to approximately 8 μm apart and shooting to the left direction. **f–h** Reproduced with permission from Ref. [253]. Copyright 2006, Institute of Physics Publishing. **i** The switch-shaped SiNW arm, driven by a vector Lorentz force, can swiftly bend downward to contact the bottom electrode or retract to overcome van der Waals forces, thereby closing the circuit. **j, k** A Micron-scale air gap ensures excellent electrical isolation while achieving an ultra-low operating voltage of less than 0.2 V. **i–k** Reproduced with permission from Ref. [65]. Copyright 2024, American Chemical Society. **l** Guiding growth of free-standing 3D silicon nanohelices (SiNHs) for NEMS resonators. **m** Vibration amplitude peaks for typical swaging back–forth resonant mode, and **n** the corresponding vibration SEM images. **l–n** Reproduced with permission from Ref. [184]. Copyright 2020, American Chemical Society

In our recent work, we demonstrate the simplest construction of slim gripper robotic structures, shaped via a single-nanowire-morphing strategy [61, 64]. The gripper can be very efficiently actuated into large-amplitude motions, powered by geometry-tailored Lorentz forces, when carrying a tunable current flow within a background magnetic field (Fig. 18a–e). The designable folding growth of ultralong and ultrathin SiNWs into single and nested omega-ring structures has been accomplished via IPSLS guiding growth mode (Fig. 18a). Then, the SiNWs can be suspended upon electrode frames and coated with silver layer to carry a passing current along geometry-tailored pathway (Fig. 18b, c). Within a magnetic field, the grippers demonstrate swift large-amplitude maneuvres of grasping (Fig. 18d), flapping (Fig. 18e) and twisting of microscale objects, as well as high-frequency or even resonant vibrations for a reliable releasing of carried payloads. Further on, more advanced double-hand collaborations and on-site LED unit picking and lighting are also demonstrated for the first time. This work provides a powerful platform to rapidly design, fabricate and testify a rich set of bionic microscale robotic structures and functionalities.

Furthermore, we developed the smallest, mechanical slingshots by using highly resilient and geometry-tailored ultrathin IPSLS SiNWs as elastic medium [253]. These elastic NW slingshots can store 10 times more strain energy than that in straight ones under the same pulling force, which can overcome the sticky van der Waals force at the touching interfaces for the reliable releasing of tiny payload units. The meandering SiNW were first grown on a planar surface (Fig. 18f), with desired layout, and then mounted upon standing pillar frames (Fig. 18g). The unique design of self-hooking structure allows for a facile and reliable assembly, loading and shooting maneuver of microsphere payloads. The microscale SiO_2_ spheres with diameter of ~ 5 μm were picked up by the slim fiber tip, and placed on the proximity of SiNW slingshot. After aligning the fiber tip to the shooting direction and placing the microsphere onto the center cockpit, the optical fiber is pulled back to desired distance for accumulating sufficient mechanical energy, relying on the high adhesive force and frictional dragging between the optical fiber and the SiNW spring. The detaching of spring from fiber tip to trigger slingshot shooting was accomplished by increasing the fiber contact angle or applying a sudden drag (Fig. 18h). This miniaturized mechanical NW slingshot could find potential applications in NEMS sensing, biological delivery or medicine therapy treatment.

We further demonstrate a novel nanowire-based nanoelectromechanical (NW-NEM) switch structure featuring an ultra-low operating voltage (Fig. 18i–k) [65]. Unlike conventional NEM switches driven unidirectionally by electrostatic attraction, the NW-NEM switch is bidirectionally actuated by Lorentz forces. This unique mechanism enables the use of large air gaps to achieve superior electrical isolation and allows the NW-NEM switch to effectively overcome adhesion forces, ensuring a reliable return to the OFF state. Remarkably, the NW-NEM switch achieves a record-low operating voltage of less than 0.2 V, significantly outperforming traditional NEM switches, which typically require high operating voltages in the range of tens of volts [254, 255].

In addition to the above in-plane growth of SiNW for flexible devices, the IPSLS mode can also be used to guide the growth of free-standing 3D silicon nanohelices (SiNHs) for NEMS resonators [184]. The sidewall grooves were first formed by simple Bosch-etching into Si wafer, using polystyrene sphere (PS) array as a template. Then, the indium catalyst droplets move around the bamboo-like cylinders in a helical fashion, while consuming precoated a-Si thin film to produce crystalline Si nanowires on the sidewalls (Fig. 18l). At the end of each groove cycle, the droplets are enforced to linefeed/switch into the neighbor groove to continue a spiral growth of SiNHs. Finally, the SiNHs can be reliably released as freestanding units to serve as vibrational resonators when treated by hydrofluoric solution.

The standing SiNHs can be electrically driven and excited to distinct resonant modes at different frequencies. For instance, a SiNH unit with 7.5 period of spiral cycles is chosen and approached by a tungsten probe in an SEM chamber with the experimental setup. The probe is kept ∼1 μm apart from the tip of SiNH, applying an alternating voltage of 5 V amplitude with frequency ramping from 1 kHz to 10 MHz. Three resonant peaks located at 0.42, 1.246, and 1.34 MHz were identified in the plots of vibration amplitude against the frequency, which can be assigned to the swaging of the standing SiNH in back–forth manners, left–right manners and up–down stretching mode vibrating along the z-axis direction, respectively. For example, Fig. 18m shows the typical back–forth manner resonant mode at peak at 0.42 MHz, where the amplitudes can be extracted from its corresponding vibration SEM images presented in Fig. 18n.

### Other Si-Based Flexible Applications

**Fig. 19:**
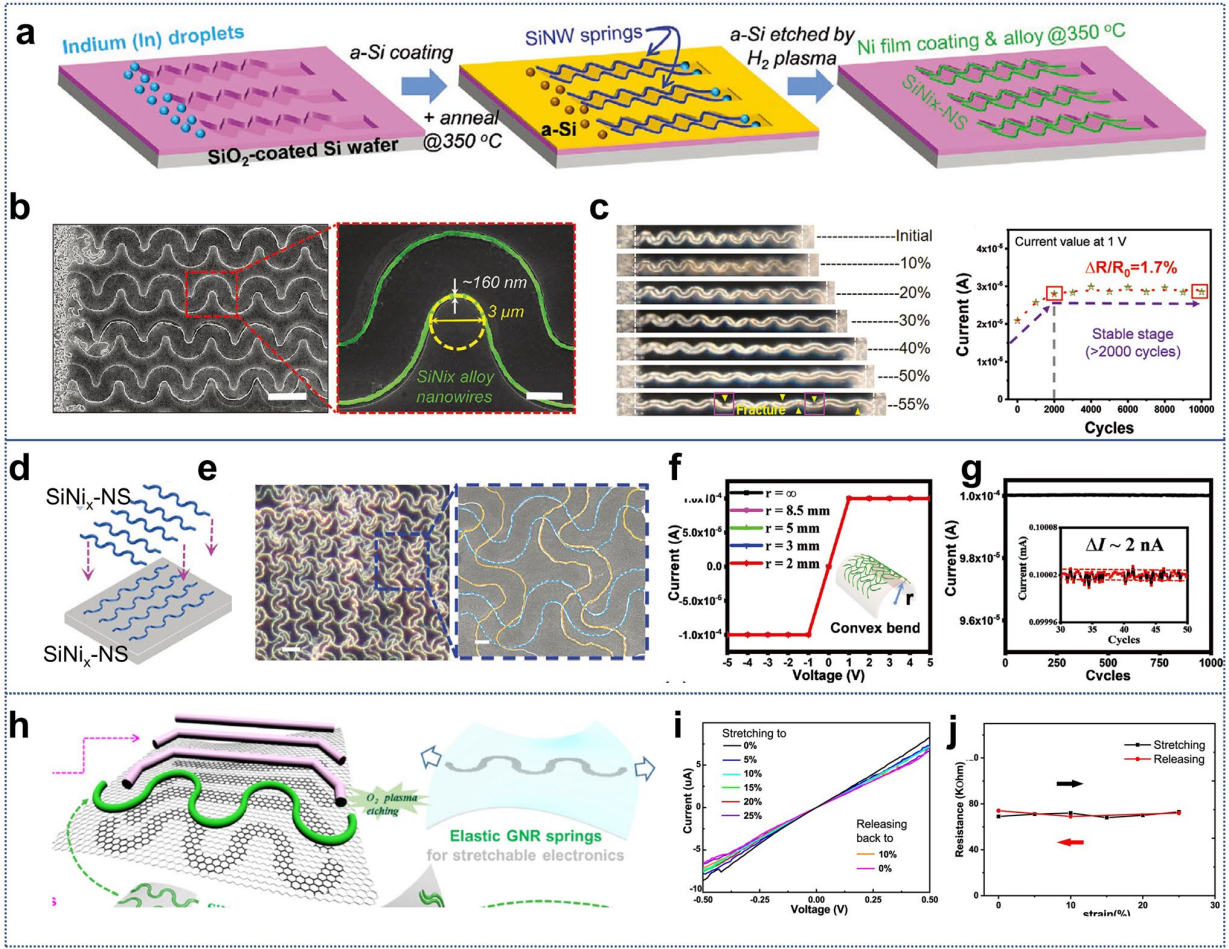
**a** Fabrication procedure of the SiNix-NS, starting from the sample surface patterning, guided growth of SiNW channels via IPSLS, Ni layer coating to a low temperature alloy-forming annealing process. **b** The typical SEM images of the as-grown SiNix-NS arrays, with a mean diameter of 160 nm and very short interconnection distance (down to ≈3 μm). **c** Microscopic images of the geometry evolution of a SiNi*x*-NS interconnection, transferred onto PDMS substrate, under gradually increasing stretching from initial to 55%; current variation recorded at 1 V bias with the increase of stretching cycles (to 15% strain) from initial to 10,000 times. **a–c** Reproduced with permission from Ref. [182]. Copyright 2021, John Wiley and Sons. **d** Schematic illustration of the stacked SiNix-NS, **e** The optical images of the mutual crossed spring-shaped Si NWs. **f, g** the I–V curves of the wavy or spring SiNi-NWs under different convex bending radii and the corresponding transport current variations under 3 V bias for repetitive 1000 bending cycles. **d–g** Reproduced with permission from Ref. [256]. Copyright 2023, John Wiley and Sons. **h** NWL patterning of a single graphene sheet into parallel channels or elastic serpentine spring. Stretching tests of the geometry-engineered GNR springs. **i** shows the current–voltage (IV) characteristics of the GNR springs under different stretching strains. **j** presents the stable GNR springs’ resistance under repetitive stretching to 25% and releasing back. Reproduced with permission from Ref. [67]. Copyright 2019, Springer Nature

High-density integration of elastic and durable interconnections is the key capability for stretchable electronics applications in biosensing, skin-mimetic electronics and flexible displays. In order to dissipate the stress accumulation at the boundary edge, a geometry scale factor $r=\frac{R}{w}\sim \frac{({L}_{\text{iti}}-2w)/2}{w}\ge 10$ is required, where *R* is the bending curvature radius of the spring channel, *w* is the width of the spring interconnector, *L*_iti_ is the spacing between the rigid-islands. For high-density integration of the stretchable electronics, *L*_iti_ is rapidly scaling down to < 20 μm, which thus requires the *w* < 900 nm. Unfortunately, such narrow spring interconnectors are difficult to pattern via conventional photolithography (for flat-panel display technology, the spatial resolution is limited to 2 ~ 3 μm), except the use of inefficient electron beam lithography (EBL) or nanoimprint lithography (NIL) technologies that are not applicable for large area stretchable electronics. In addition, the conductive interconnections among the islands must be highly elastic and robust to sustain or dissipate large stretching strain. Therefore, we demonstrate a large-scale and precise integration of highly conductive nickel silicide nanospring (SiNi_*x*_-NS) array (Fig. 19a–c) [182]. The SiNW springs are first fabricated out of an IPSLS guided growth, and are subsequent alloy-formed with Ni at 350 °C to boost the channel conductivity over 4 orders of magnitude (to 2 × 10^4^ S cm^−1^). Thanks to the nanoscale diameter of these spring SiNi_*x*_ interconnectors, the elastic geometry engineering can be accomplished within a very short distance (down to ~ 3 μm). Deployed over soft PDMS thin film substrate, the SiNi_*x*_-NS array demonstrate an excellent stretchability that can sustain up to 50% stretching and for 10,000 cycles (at 15%). A rather stable value of Δ*R*/*R*_0_ = 1.7% was calculated during the following 10^4^ cycles under 1 V bias. Therefore, this work paves the new way to integrate stable and robust inorganic interconnections for high-density and high-performance electronics for health monitoring, displays and on-skin electronic applications, based on the mature and rather reliable Si thin film technology.

Interestingly, these conductive SiN_x_-NS can be orthogonally-stacked into a nanowire network for flexible and transparent thin film application [256]. The orderly SiNW arrays grown via IPSLS mechanism were reliably transferred and cross-stacked upon flexible polyimide (PI) films to weave a quasicontinuous network with designable orthogonal-stacking layout that guarantees simultaneously a continuous conductive pathway of crossed NWs and a high overall transparency (Fig. 19d, e). To enhance the conductivity of the network, the SiNWs on PI substrate were coated with Ni thin film and annealed to transform into highly conductive SiNix-NS, which are directly connected to each other at the crossing points, without the use of any other metal electrodes. The SiNix-NS network demonstrate high flexibility because of the elastic spring design of the silicide NW channels, a moderately equivalent sheet resistance of 130 Ω sq^−1^ and a durable flexibility that can sustain repetitive bending to 2 mm radius for > 1000 cycles (Fig. 17f, g). Thanks to the minimal stacking SiNW number of ${N}_{\text{stk}}=2$, the SiNix-NS show an impressive transmittance of ~ 90%. These results indicate a new scalable and economic network formation or integration strategy of highly conductive and durable inorganic NWs for future flexible electronics, displays and bio-interfaced sensors.

These stretchable SiNWs can also be used as a mask for patterning other materials for soft electronics. For example, we developed a precise and scalable nanowire lithography technology that enables reliable batch manufacturing of ultralong graphene nanoribbon (GNR) arrays with programmable geometry and narrow width down to ~ 50 nm [67]. First, the PMMA solution was spin-coated onto the source substrate where SiNW spring arrays are grown. The substrate was then immersed in 4% HF solution to separate the SiNW-loaded PMMA film from the source substrate. Then, the PMMA film was flipped and placed on the target substrate with graphene on top, forming a SiNW/PMMA/graphene stack that was annealed subsequently at 150 °C for 2–3 min to enhance the adherence of the whole structure. After that, the transferred SiNWs served as nanoscale shadow masks during an oxygen plasma reactive ion etching (Fig. 19h). This thus etched off the exposed PMMA layer and GNRs, leaving only the segments protected by the SiNW masks. Finally, the PMMA and SiNWs were removed by using hot acetone at 60 °C.

The electrode connection configuration has been illustrated in the inset of Fig. 19h, where two Au/silver (Ag) paster pads are connected by 2–3 GNRs with a separation of ~ 50 μm. Thanks to the capability of the geometry design, the GNRs can be predesigned and engineered into elastic 2D springs to achieve an outstanding stretchability up to 25%, without obvious resistance change under repetitive stretching and releasing back (Fig. 19i, j). This convenient NW lithography technology thus holds a strong promise to establish a general strategy for integrating various nano-channel patterns of 2D materials for developing flexible/stretchable electronics and sensors.

## Conclusions and Outlook

In this review, we have explored the significant advancements in the integration of Si materials into flexible electronics, emphasizing innovative strategies that overcome the intrinsic stiffness of Si. From the use of 3D bulk Si to 2D thin films and 1D SiNWs, a series of structural design approaches have been employed to enhance the mechanical flexibility of Si-based devices. The creation of discrete silicon islands structures on flexible substrates allows bulk c-Si devices to bend freely under mechanical stress without compromising their electronic performance. Further innovations, such as thinning bulk Si or the top layer of SOI wafers to the micro- or nanometer scale, have significantly improved flexibility. Additionally, the geometric shaping of SiNWs, fabricated through advanced methods such as VLS and IPSLS, provides a pathway to achieve ultra-flexible structures, further enhancing the performance and versatility of Si-based flexible electronics.

Leveraging these geometry engineering strategies, a wide range of Si-based soft electronic devices with remarkable biocompatibility, long-term stability, and high performance have been developed, leading to diverse applications in fields, such as sensors, cellular signal detection, robotics, and brain-machine interfaces. However, despite these advancements, flexible electronics face inherent limitations. Trade-offs in performance, durability, and cost often accompany their design, posing challenges that need to be addressed. Understanding and mitigating these trade-offs is critical for optimizing functionality and realizing the full potential of flexible electronics across diverse applications. To further advance Si-based flexible electronics, several key challenges must be addressed:

1. *Integration of Hard Si Electronics with Stretchable Conductive Interconnectors:* While innovative structural designs such as Si islands and thin films have facilitated the development of high-performance Si-based devices, the integration of these components into high-density systems for complex electronic architectures remains a significant challenge. The precise fabrication and positioning of stretchable conductive interconnects are essential to ensure reliable electrical connections and performance, particularly for applications requiring both high density and flexibility.
2. *Development of Multi-Functional Soft Devices:* One of the exciting directions in the field is the creation of multi-functional soft electronic devices. Si materials possess diverse properties such as piezoresistivity, temperature sensitivity, and photosensitivity, making them suitable for integration into multi-functional systems. When used in FETs, Si channels exhibit remarkable sensitivity to ions, charge variations, and environmental stimuli, enabling detection of gases, biological markers, and other physiological signals. However, effectively integrating these diverse functionalities into a single chip while maintaining device performance and flexibility remains a significant challenge.
3. *Achieving Ultra-Flexible and High Stretchability for Real-World Applications:* While current Si-based flexible electronics primarily excel in bending flexibility, they fall short in achieving the high stretchability required for applications such as soft human tissues, including skin and organs. These tissues demand devices that can stretch beyond 30% without failure, which is a key challenge for achieving seamless integration into wearable or implantable electronics. SiNWs, due to their unique geometry, hold great potential for meeting this requirement. The IPSLS-guided growth strategy, which allows for the fabrication of SiNWs in arbitrary shapes, could be pivotal in developing ultra-stretchable devices capable of meeting these needs.

The continued development of Si-based flexible electronics will rely on overcoming these challenges, particularly in enhancing the integration of Si-based materials with stretchable interconnects and advancing multi-functional device architectures. These efforts will enable the creation of next-generation, highly flexible and stretchable electronic systems with broad applications in areas such as healthcare, environmental monitoring, wearable technology, and beyond. As research progresses, new breakthroughs in materials, fabrication techniques, and device integration are expected to further enhance the capabilities of Si-based flexible electronics, driving the development of advanced technologies that will have a profound impact on everyday life and contribute to the advancement of various scientific and industrial fields.
